# A Combined Magnetic and Mössbauer Spectroscopic Study of High-Spin Half-Sandwich Fe(I) Complexes in Monomeric and Polymeric Configurations

**DOI:** 10.1021/acs.inorgchem.5c03729

**Published:** 2025-11-18

**Authors:** Katharina Münster, Dirk Baabe, Jan Raeder, Benjamin Kintzel, Oluseun Akintola, Michael Böhme, Winfried Plass, Marc D. Walter

**Affiliations:** † Institut für Anorganische Und Analytische Chemie, 26527 Technische Universität Braunschweig, Braunschweig 38106, Germany; ‡ Institut für Anorganische Und Analytische Chemie, 9378 Friedrich-Schiller-Universität Jena, Jena 07743, Germany

## Abstract

The reduction of the iron­(II) half-sandwich precursors [Cp′FeN­(R)­(SiMe_3_)] (*R* = SiMe_3_, 2,6-di-*iso*-propylphenyl (dipp)) with KC_8_ yields the one-dimensional polymeric iron­(I) complexes {[Cp′Fe­(N­(SiMe_3_)_3_)]­K}_n_ (**4**) and {[Cp′Fe­(N­(dipp)­(SiMe_3_))]­K}_n_ (**5**). Addition of 18-crown-6 breaks up these chains and affords the monomeric salts [Cp′Fe­(N­(SiMe_3_)_2_)]­[K­(18-crown-6)­(thf)_2_] (**6**) and [Cp′Fe­(N­(dipp)­(SiMe_3_))]­[K­(18-crown-6)­(thf)_2_] (**7**). All four compounds were structurally characterized by single-crystal X-ray diffraction analysis and adopt an *S* = 3/2 spin ground state. Zero-field ^57^Fe Mössbauer spectra uncover slow magnetic relaxation at low temperatures in each complex. Complementary CASSCF/NEVPT2 calculations reveal an *S* = 3/2 ground-state Kramers doublet that is ca. 100 cm^–1^ below the first excited state. These findings are confirmed by results from DC susceptibility and magnetization experiments. The slow relaxation of magnetization is further characterized via AC susceptibility measurements, unveiling detectable slow relaxation of magnetization at zero external field for **4**–**6**. Complex **4** exhibits an unusual field dependence of the quantum tunneling of magnetization, akin to exchange-bias effects, which are commonly observed in lanthanide dimers.

## Graphical abstract

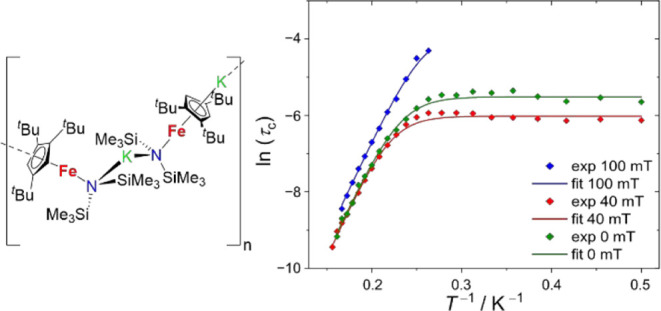

## Introduction

The earth-abundant, inexpensive, and essential element, iron is an especially attractive transition metal for catalysis, small molecule activation, and materials science.^1–3^ Since the landmark discovery of ferrocene [(η^5^-C_5_H_5_)_2_Fe],^4,5^ cyclopentadienyl (Cp) ligands have become ubiquitous in organometallic complexes, resulting in various sandwich and half-sandwich complexes featuring Cp derivatives.^6^ To (kinetically) stabilize low-valent, low-coordinate species, bulky ligands are particularly effective at providing the necessary steric protection at the metal atom. In this vein, several low-coordinate Fe­(I) compounds bearing sterically encumbered amido, alkyl, cyclopentadienyl or *N*-heterocyclic carbene (NHC) ligands have been reported (Figure 1). These ligands not only shield the iron atom sterically but also reinforce its low-valent state through strong σ and/or π-donation.

**Figure 1:**
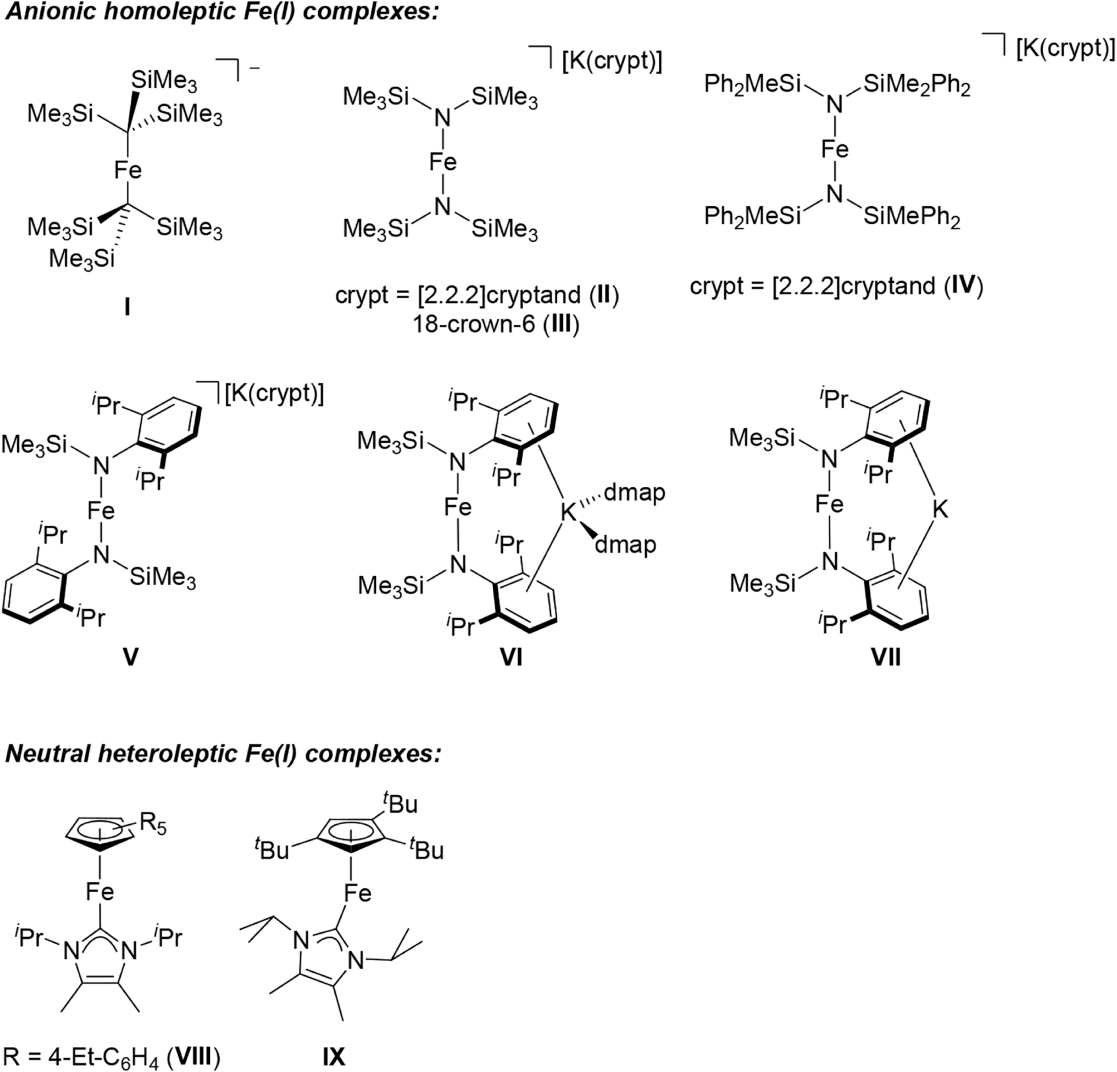
Examples of low-coordinate Fe­(I) complexes.^7–15^

We recently reported on the characterization of three related Fe­(II) half-sandwich amido complexes [Cp’Fe­{N­(SiMe_3_)­(R)}] (*R* = SiMe_3_, 2,6-di-*iso*-propylphenyl, ^*t*^Bu) employing ^57^Fe Mössbauer spectroscopy and magnetic (DC and AC) susceptibility measurements which revealed significant zero-field splitting (ZFS) and slow paramagnetic relaxation in those systems.^16^ Remarkably, only [Cp’Fe­{N­(^*t*^Bu)­(SiMe_3_)}] exhibits slow paramagnetic relaxation in its AC magnetic susceptibility even in the absence of an applied static magnetic field, so far an unprecedented behavior for pseudolinear Fe­(II) complexes. This is because from the very beginning of research on single molecule magnets (SMM), large axial magnetic anisotropy (parametrized as axial ZFS parameter *D*) was identified as a crucial ingredient for achieving large blocking temperatures (*T*_B_),^17,18^ which are required to make such molecules feasible for the designated applications in data storage^19^ and quantum computing.^20^ To circumvent the need for precise alignment and exchange coupling in multinuclear SMMs, the research focus turned to single-ion magnets (SIMs), in which all anisotropy resides on one metal center.^21^ An obvious way to maximize axial magnetic anisotropy in SIMs is to combine a strongly axial coordination environment - most simply realized in linear geometries - with an appropriate valence-electron configuration (*e*.*g*., d^7^ for transition-metal SIMs or f^9^ for lanthanide SIMs). Indeed, such strategies spawned the so far largest reported anisotropy barriers (*U*_eff_) for lanthanide^22,23^ as well as transition metal systems.^24^ Comparable examples based on Fe­(I) include [Fe­{C­(SiMe_3_)_3_}_2_]^−^ (**I**; C–Fe–C 179°),^7^ [Fe­{N­(SiMe_3_)_2_}_2_]^−^ (N–Fe–N 180° (**II**) and (**III**)),^10^ [Fe­{N­(SiMePh_2_)_2_}_2_]^−^ (N–Fe–N 178.1° (**IV**)),^11^ [KFe­{N­(SiMe_3_)­(2,5-^***i***^Pr_2_C_6_H_3_)}_2_]^−^ (N–Fe–N 177.79° (**IV**))^13^ and [(η^5^-C_5_(4-Et-C_6_H_4_)_5_)­Fe­(IPr_2_Me_2_)] (**VIII**; Cp–Fe–C 171°) (Figure 1).^14^ However, once *U*_eff_ is sufficiently large, magnetic relaxation is predominantly driven by quantum tunneling^25–29^ and vibrationally assisted relaxation processes.^30–33^ The former is strongly coupled to the purity of involved electronic states and hence local symmetry of the ligand field, while the latter also depends on the rigidity and symmetry of the molecule as well as the overall solid-state structure. Within this framework, investigating complexes with large magnetic anisotropy and comparable coordination environments both in molecular and polymeric forms appears highly desirable.

In this contribution, we build on our previous report^16^ by detailing the synthesis and characterization of Fe­(I) compounds obtained by reduction of the corresponding Fe­(II) precursors [Cp’Fe­{N­(SiMe_3_)_2_}] (**2**) and [Cp’Fe­{N­(dipp)­(SiMe_3_)}] (**3**; dipp = 2,6-di-*iso*-propylphenyl). This study comprises of structural details, zero-field ^57^Fe Mössbauer spectroscopy and magnetic susceptibility measurements in combination with *ab initio* calculations.

## Results and Discussion

### Synthesis of Fe­(I) Amido Species

The salt metathesis reaction of [Cp’Fe­(μ-I)]_2_ (**1**) with lithium amides [Li­{N­(SiMe_3_)_2_}­(OEt_2_)]_2_ and [Li­{N­(dipp)­(SiMe_3_)}]_2_ yields the two-coordinate, high-spin Fe­(II) half-sandwich complexes **2** and **3**.^16^ Reduction with KC_8_ furnishes the Fe­(I) coordination polymers **4** and **5** in good isolated yields. These polymeric species can be converted into the monomeric Fe­(I) compounds **6** and **7** either by adding 18-crown-6 after the reduction or by carrying out the reduction in its presence, affording **6** and **7** in good to moderate yields (Scheme 1). Upon prolonged exposure to dynamic vacuum the coordinated THF ligands attached to the cation [K­(18-crown-6)­(thf)_2_]^+^ can be removed. All complexes are moisture- and air-sensitive. Furthermore, slow degradation of these complexes is observed over about two months in the solid state, even when stored at –30 °C under an inert atmosphere. Nevertheless, they display reasonable thermal stability, decomposing or melting between ca. 70 and 120 °C (see the Experimental Section for details). They are soluble in THF and Et_2_O but are only sparely soluble in *n*-pentane, *n*-hexane and benzene.

**Scheme 1:**
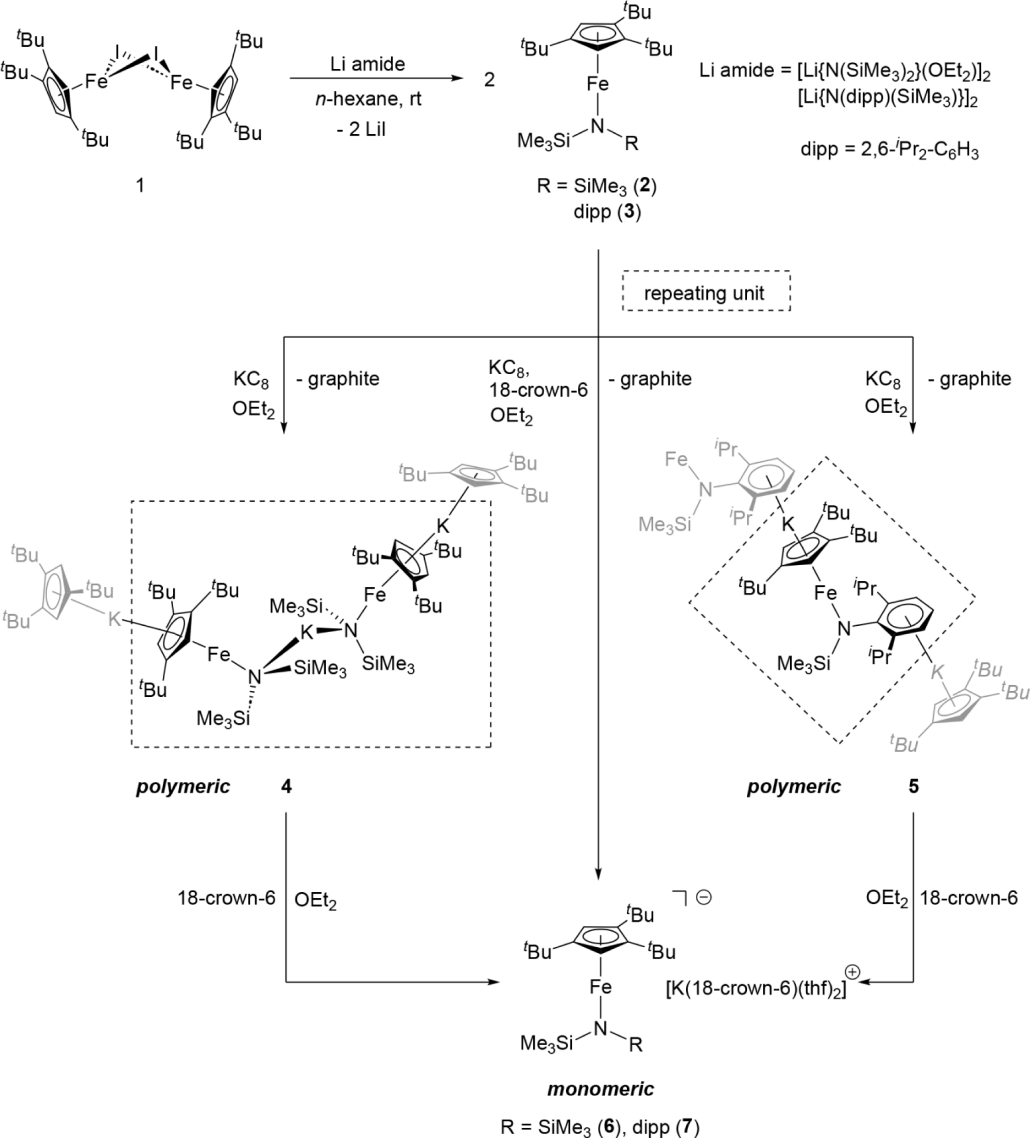
Synthesis of Polymeric and Monomeric Fe­(I) Amido Complexes **4**–**7** from Literature-Reported Fe­(II) Amido Species **2** and **3** ^16^

### ^1^H NMR Spectroscopy

Complexes **4**–**7** exhibit strongly broadened ^1^H NMR resonances in THF-*d*_8_, as expected for paramagnetic Fe­(I) d^7^ complexes (Table 1). In the N­(SiMe_3_)_2_–substituted derivatives **4** and **6**, every resonance except those for the Cp-bound protons could be observed and assigned. In the dipp-functionalized complexes **5** and **7**, the aromatic phenyl C–H resonances are unobserved, whereas the Cp-bound protons in **5** appeared as a broad resonance. Compared to the Fe­(II) precursors **2** and **3**, the *tert*-butyl proton resonances are shifted significantly downfield. Addition of 18-crown-6 has only a minimal effect on these *tert*-butyl proton chemical shifts.

**Table 1:** ^1^H NMR Chemical Shifts *δ* (ppm) of Amido-Substituted Iron Compounds **2**–**7** ^d^

	1 × ^*t*^Bu (9 H)	2 × ^*t*^Bu (18 H)	2 × Cp’-H
**2** ^a^ ^34,35^	–42.6 (700)	–30.3 (1050)	^c^
**3** ^a^ ^16^	–22.8 (650)	–33.2 (920)	^c^
**4** ^b^	–0.4 (330)	–13.1 (860)	^c^
**5** ^b^	–5.1 (300)	–11.6 (320)	31.6 (1200)
**6** ^b^	2.9 (500)	–12.0 (450)	^c^
**^–1^** ^b^	–4.9 (300)	–11.4 (300)	^c^

a Recorded in C_6_D_6_ solution at 298 K.

b Recorded in THF-*d*_8_ solution at 298 K.

c No resonance is observed at room temperature.

d The values in parentheses are the full widths at half maximum (ν_1/2_ in Hz).

### X-ray Crystallography

Single crystals suitable for X-ray diffraction analysis of the Fe­(I) complexes **4**–**7** were successfully obtained (see Experimental Section for details). It should be noted, that attention to the crystallization conditions is crucial, since these complexes tend to precipitate as polycrystalline powders from cooled saturated solutions. We found that precooling the sample holder to –30 °C before harvesting the crystals from their mother liquor, and keeping the system at that temperature until immediately prior to microscope inspection and mounting on the diffractometer, significantly improved crystal quality. Molecular structures are shown in Figures 2–4. Single-crystal X-ray diffraction data on crystals of complex **6** were measured at *T* = 100 and 80 K. In both cases, the compound was found to adopt space group *P*1̅, with slightly different unit-cell dimensions but virtually identical bond parameters (see Tables S1 and S2 in the Supporting Information). All complexes contain one molecule per asymmetric unit, except **4**, whose asymmetric unit contains two crystallographically independent Cp–Fe–N chain fragments with marginal structural differences. Selected bond lengths and angles are listed in Tables 2 and S2 (Supporting Information).

**Figure 2:**
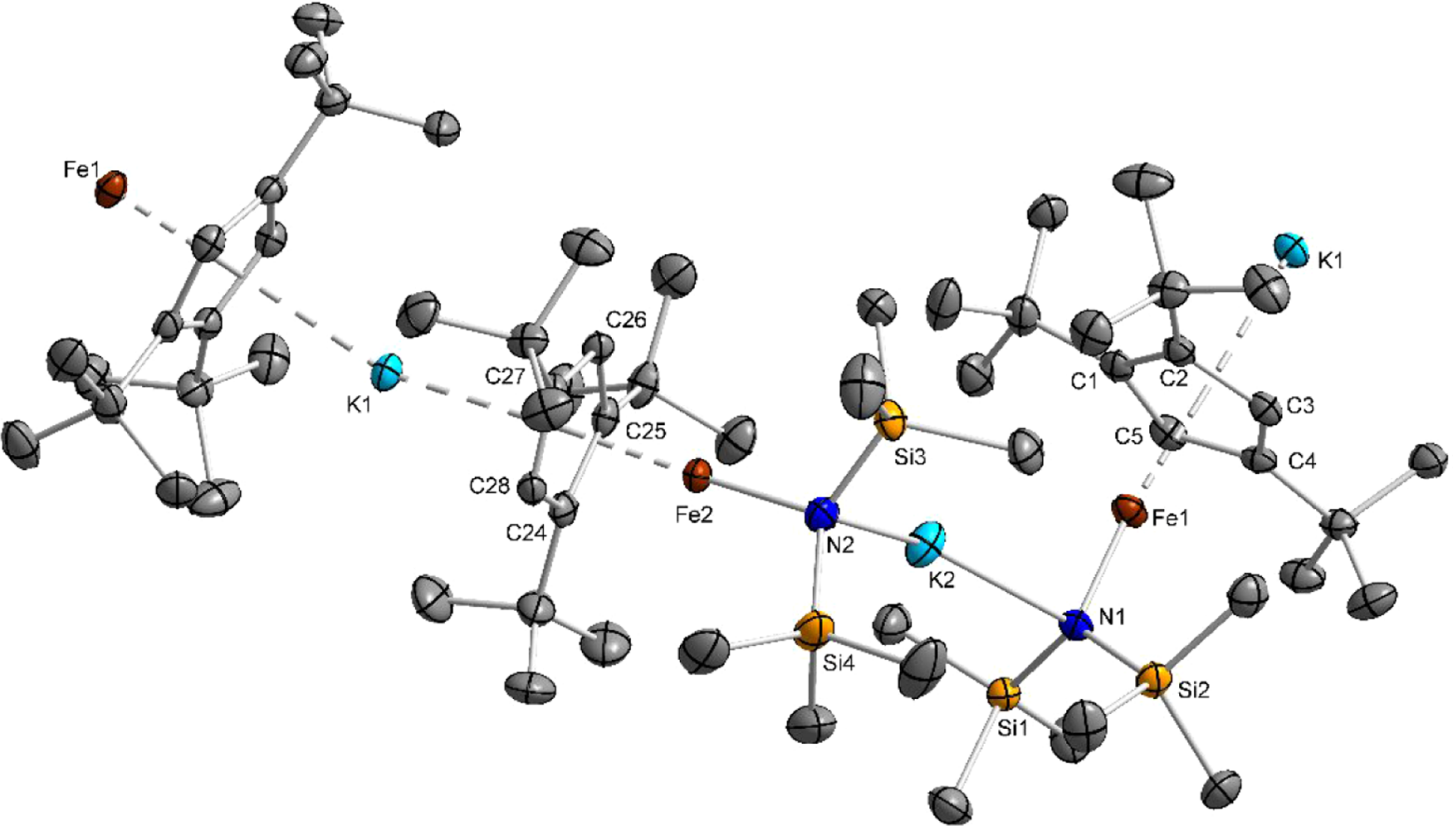
Diamond plot of a cutout from the polymeric solid-state structure of **4** at *T* = 100 K. Ellipsoids are drawn at the 50% probability level. Hydrogen atoms are omitted for clarity.

**3:**
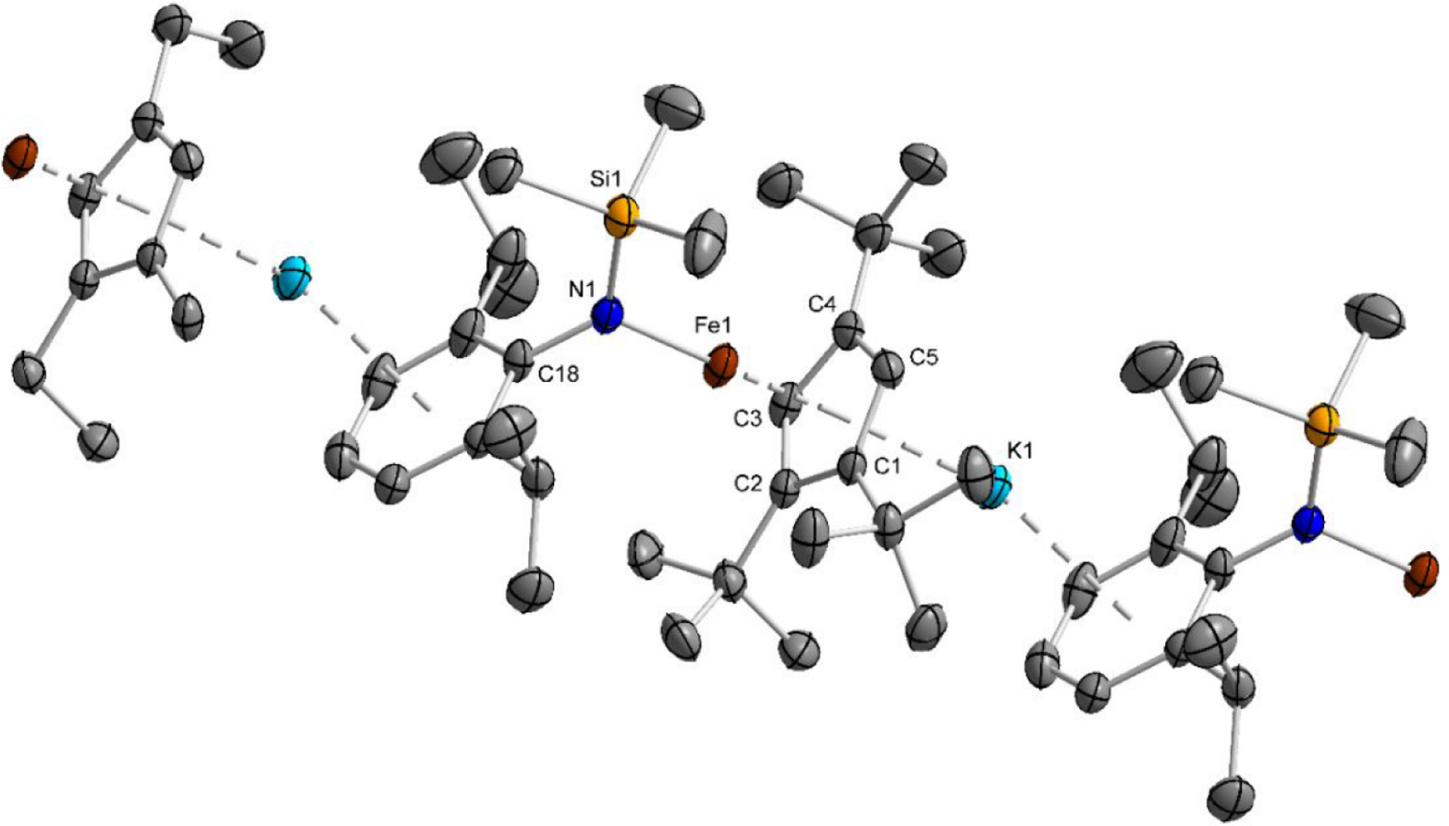
Diamond plot of a cutout from the solid-state structure of **5** at *T* = 100 K. Ellipsoids are drawn at the 50% probability level. Hydrogen atoms are omitted for clarity.

**Figure 4:**
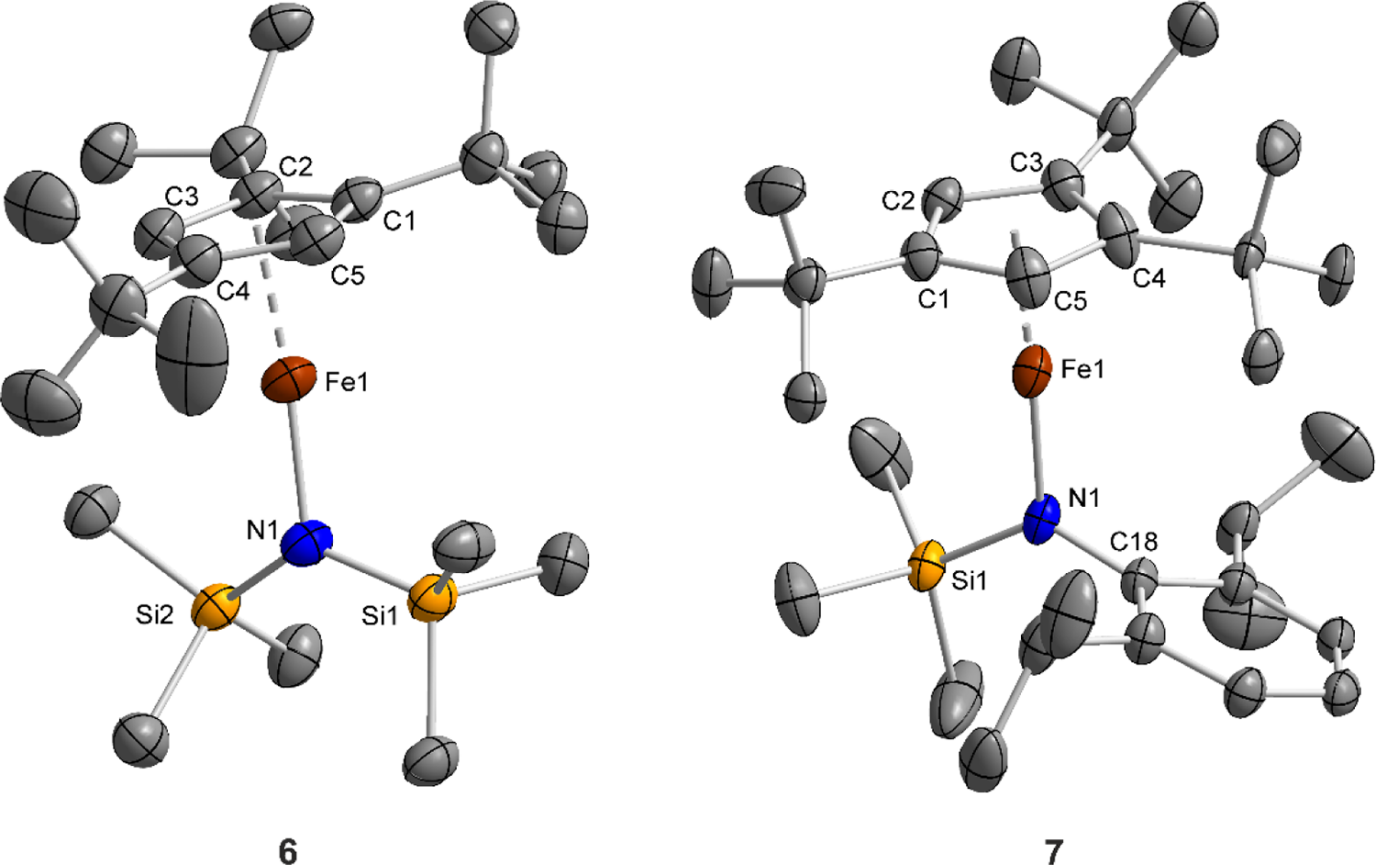
Diamond plots of the molecular structures of **6** and **7** in the solid state at *T* = 100 K. Ellipsoids are drawn at the 50% probability level. Hydrogen atoms and the chelated counter cations [K­(18-crown-6)]^+^ are omitted for clarity.

**Table 2:** Selected Bond Lengths (Å) and Angles (°) for iron­(I) Complexes **4**–**7** at *T* = 100 K^a^

	**4**	**5**	**6**	**7**
Cp_cent(A)_–Fe1(2)	1.930(1)/1.913(1)	1.908(1)	1.908(1)	1.900(1)
Fe1–C1(24)	2.233(4)/2.209(4)	2.255(2)	2.238(2)	2.324(1)
Fe1–C2(25)	2.291(4)/2.226(4)	2.200(2)	2.247(2)	2.280(2)
Fe1–C3(26)	2.323(4)/2.284(4)	2.238(2)	2.259(2)	2.226(2)
Fe1–C4(27)	2.319(4)/2.347(4)	2.324(2)	2.298(2)	2.198(2)
Fe1–C5(28)	2.235(4)/2.273(4)	2.298(2)	2.261(2)	2.246(2)
Fe1(2)–N1(2)	1.953(3)/1.944(4)	1.931(2)	1.934(2)	1.943(2)
K2–N1(2)	2.911(4)/2.955(4)	-	-	-
K1–Cp_cent(1–5/24–28)_	2.798(1)/2.804(1)	2.778(1)	-	-
K–Ph_cent_	-	2.909(1)	-	-
Fe···Fe	7.387/9.362 (in chain) 8.852, 9.194 (between chains)	10.699 (in chain) 9.828, 10.795 (between chains)	9.856	10.853
Cp_cent(1–5/24–28)_–Fe1(2)–N1(2)	173.0(1)/172.1(2)	177.5(1)	170.7(1)	175.8(1)
Cp_cent_–K1–Ph_cent_	-	163.6(1)	-	-
N1–K2–N2	155.3(1)	-	-	-
Cp_cent_–K2–Cp_cent_	164.6(1)	-	-	-
K2–N1(2)–Fe1(2)	104.0(2)/95.8(2)	-	-	-
K1–Cp_cent_–Fe1	176.6(1)	177.4(2)		
Fe1(2)–N1(2)–Si1(4)	111.1(2)/110.7(2)	118.6(2)	112.8(2)	118.0(1)
Fe1(2)–N1(2)–Si2(3)	115.1(2)/115.1(2)	-	118.6(2)	-

a Data for 6 at T = 80 K are listed in the Supporting Information (Table S2).

X-ray diffraction analysis of the Fe­(I) complexes prepared without 18-crown-6 shows that **4** and **5** adopt polymeric architectures, but with distinct bridging motifs. In **4**, each repetition unit comprises two Cp–Fe–N moieties bridged alternately by K^+^ ions to two amide nitrogen atoms or two Cp rings, yielding helical chains whose nearest interhelix contacts are 2.14 Å apart via SiMe_3_ protons. By contrast, **5** forms linear chains of a single Cp–Fe–N unit type, with K^+^ sandwiched between Cp and phenyl rings; the shortest interchain contact (2.35 Å) involves *tert*-butyl and *iso*-propyl methyl protons. Such coordination polymers are known for metallocenes of main-group metals (Li–Cs),^36,37^ manganese^38,39^ and certain f-block species,^40^ but are only the second example in iron half-sandwich chemistry aside from the homoleptic [Fe­{N­(dipp)­(SiMe_3_)}_2_]­K, which also features amide K^+^ aryl bridges.^41^

When 18-crown-6 is present (complexes **6** and **7**), K^+^ is chelated and the polymer breaks down to discrete monomers. All four Fe­(I) species exhibit Cp_cent_–Fe distances of ca. 1.9 Å, typical for high-spin Fe (I) complexes (Table 3). The Cp_cent_–Fe–N angles, an indicator of coordination linearity, are 173° (**4**), 170° (**6**), 178° (**5**) and 176° (**7**). Compared to the 171° in the parent Fe­(II) complex **3**, linearity clearly increases upon reduction to the Fe­(I) compounds **5** and **7**. In the polymeric chains of **4**, Fe···Fe separations are 7.39 Å and 9.36 Å (versus 10.67 Å in **5**), and interchain distances are 8.85 Å and 9.19 Å (versus 9.83 Å and 10.80 Å in **5**). In the crystal structures of the monomeric complexes **6** and **7**, the nearest Fe···Fe distances expand to 9.86 Å and 10.85 Å, respectively, reflecting well-separated anions.

**Table 3:** Correlation between Spin States and Cp_cent_–Fe Distances

Compound	Oxidation state	Spin state *S*	Cp_cent_–Fe (Å)
[Cp’_2_Fe][SbF_6_][Bibr ref42]	+III	1/2	1.77
[Cp’_2_Fe][Bibr ref43]	+II	0	1.72
[Cp’FeN(SiMe_3_)(dipp)][Bibr ref16]	+II	2	1.89
[Cp’FeI(I^*i*^Pr_2_Me_2_)][Bibr ref44]	+II	2	2.00
[Cp’Fe(I^*i*^Pr_2_Me_2_)][Bibr ref15]	+I	3/2	1.85
[Cp’Fe(I^*i*^Pr_2_Me_2_)(N_2_)][Bibr ref15]	+I	1/2	1.75

### Zero-Field ^57^Fe Mössbauer Spectroscopy

Polycrystalline samples of compounds **4**–**7** were investigated by zero-field ^57^Fe Mössbauer spectroscopy in the temperature range between *T* = 5 and 60 K. The absorption spectra of all studied compounds reveal the typical line shape and temperature-dependence that indicate the presence of slow paramagnetic relaxation with correlation times (*τ*_c_) that are long compared to the Larmor precession time of the ^57^Fe nuclear magnetic moment.^45^ The spectra were analyzed using the stochastic relaxation model developed by Blume and Tjon, which assumes fluctuations of the local magnetic hyperfine field parallel to the principal component (*V*_*zz*_) of the local electric field gradient tensor.^46^

At *T* = 60 K, the Mössbauer spectra (Figures 5 and S10 and S12 in the Supporting Information) of complexes **4**–**7** exhibit remarkably similar parameters, with isomer shifts (*δ*) ranging from *δ* = 0.72 to 0.77 mm s^–1^ and quadrupole splittings (Δ*E*_*Q*_) between Δ*E*_*Q*_ = –1.82 and –2.04 mm s^–1^ (Table 4). These values are in line with those reported for other high-spin, *S* = 3/2, Fe­(I) complexes,^8,10,15,47^ an assignment that is further corroborated by our (DC) magnetic susceptibility measurements and computational studies (*vide infra*). At *T* = 5 K (Figures 6 and 7), the absorption lines are significantly broadened, and an additional component is observed in the Mössbauer spectra for all investigated complexes **4**–**7**. This leads to a somewhat greater uncertainty in their fitted spectral parameters, which now display a larger variation for the isomer shift with *δ* = 0.50 to 0.88 mm s^–1^ and quadrupole splitting with Δ*E*_*Q*_ = −1.66 to −2.30 mm s^–1^ (Table 4).

**Figure 5:**
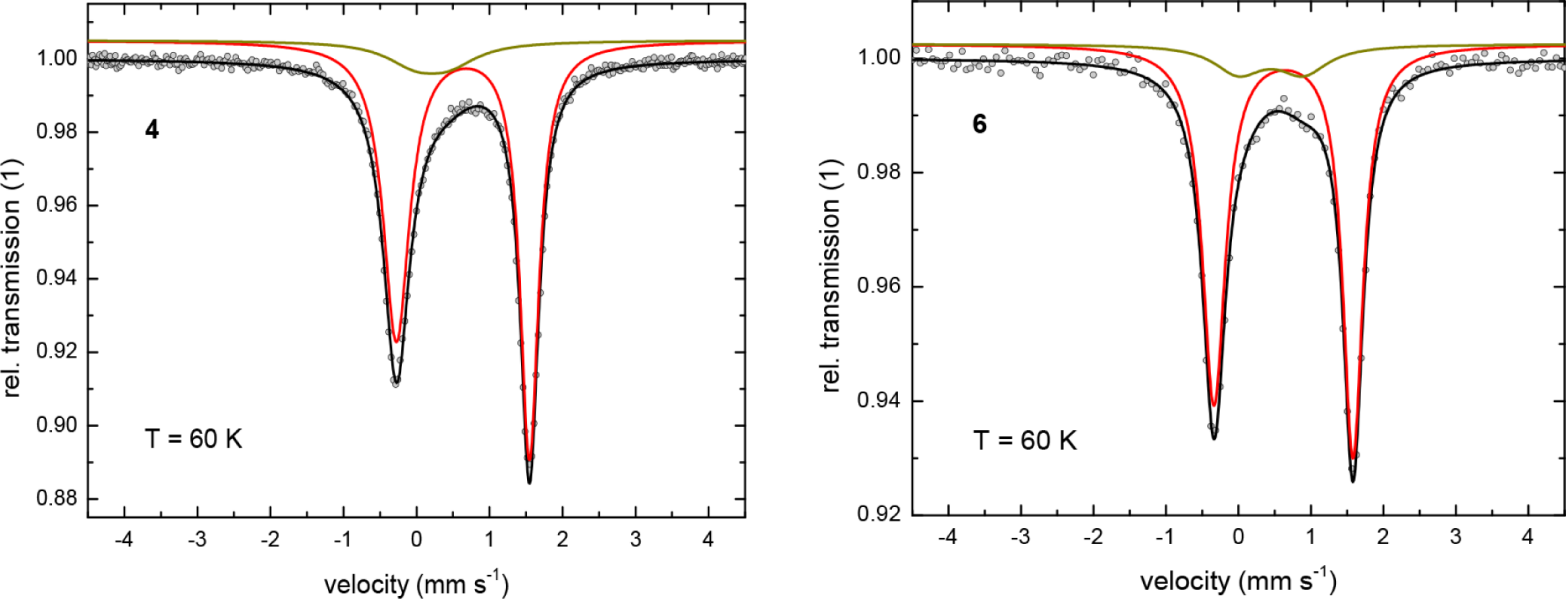
Zero-field ^57^Fe Mössbauer spectra for the chain polymer **4** and the corresponding monomer **6** at *T* = 60 K. Symbols: experimental data. Solid lines: fit with the Blume-Tjon relaxation model.^46^ The black lines represent the superposition of the two subspectra associated with compounds **4** and **6** (red), respectively, and an unidentified impurity (dark yellow). Parameters of the fits are shown in Table 4.

**Figure 6:**
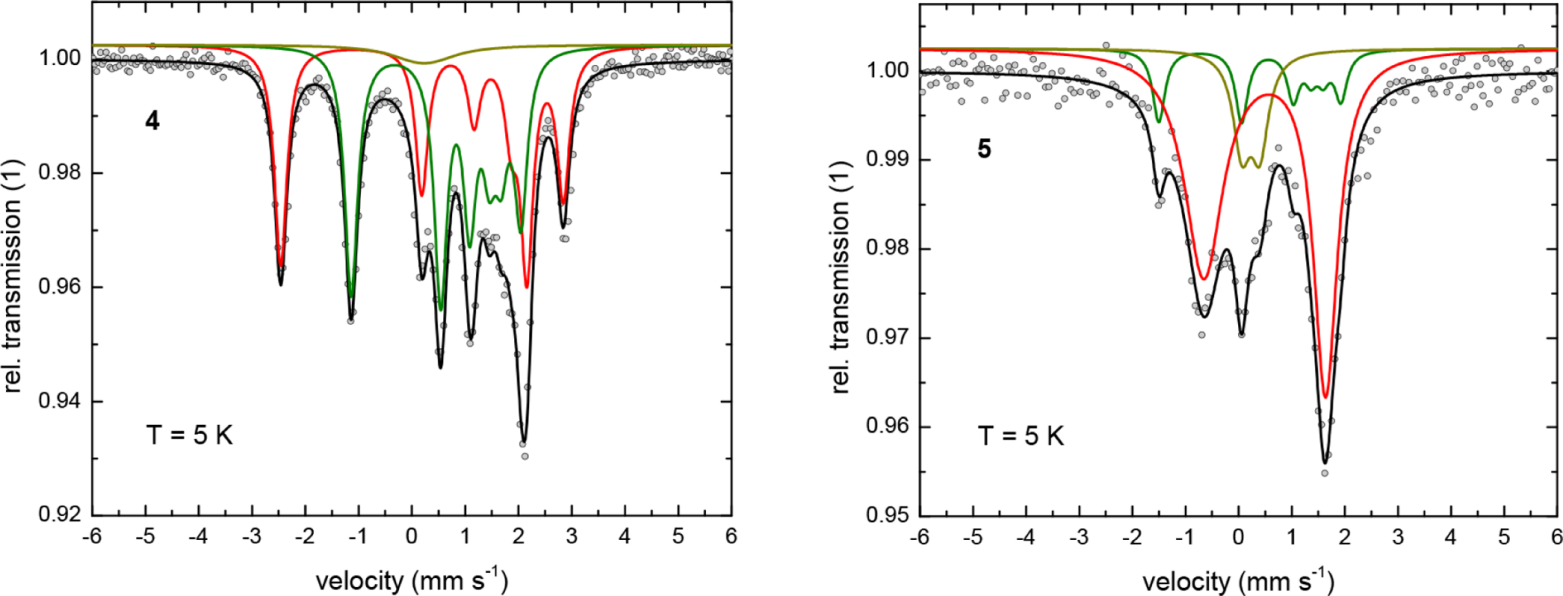
Zero-field ^57^Fe Mössbauer spectra for the chain polymers **4** and **5** at *T* = 5 K. Symbols: experimental data. Solid lines: fit with the Blume-Tjon relaxation model.^46^ The black lines represent the superposition of the subspectra associated with two nonequivalent ^57^Fe sites (red and green) in **4** and **5**, and the presence of an unidentified impurity (dark yellow). Parameters of the fits are shown in Table 4.

**Figure 7:**
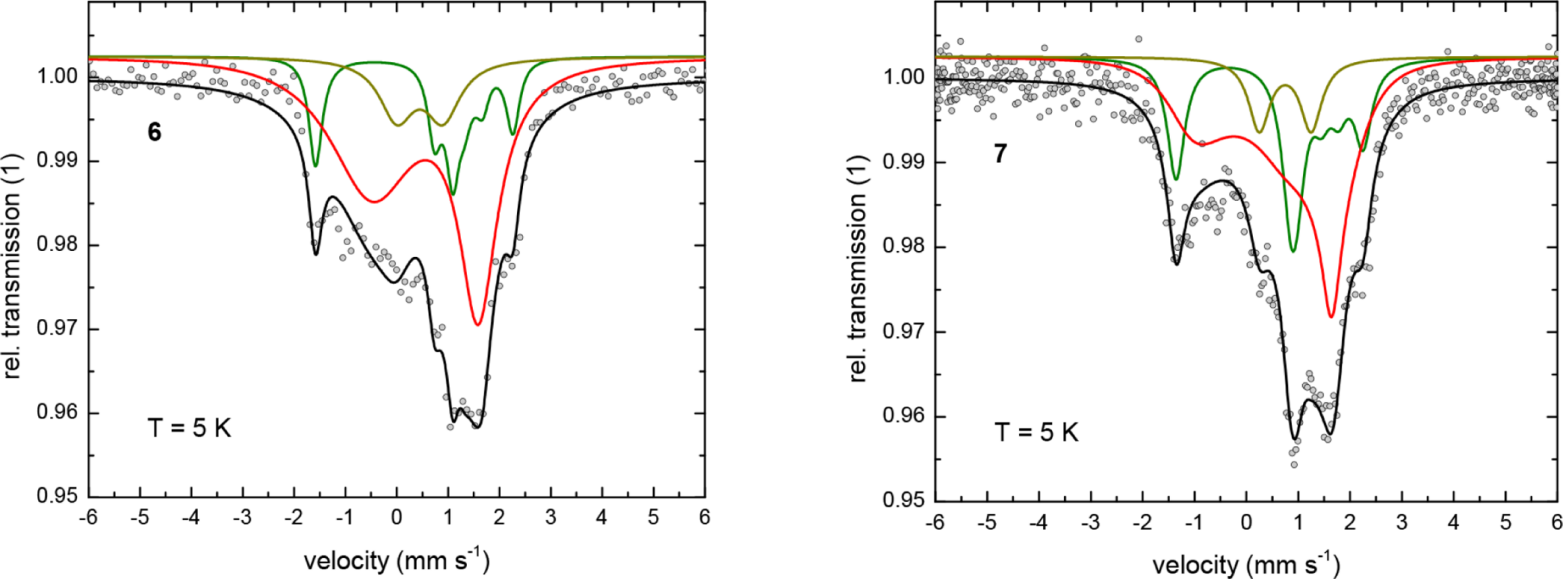
Zero-field ^57^Fe Mössbauer spectra for the monomers **6** and **7** at *T* = 5 K. Symbols: experimental data. Solid lines: fit with the Blume-Tjon relaxation model.^46^ The black lines represent the superposition of the subspectra associated with two nonequivalent ^57^Fe sites (red and green) in **6** and **7**, and the presence of an unidentified impurity (dark yellow). Parameters of the fits are shown in Table 4.

**Table 4:**
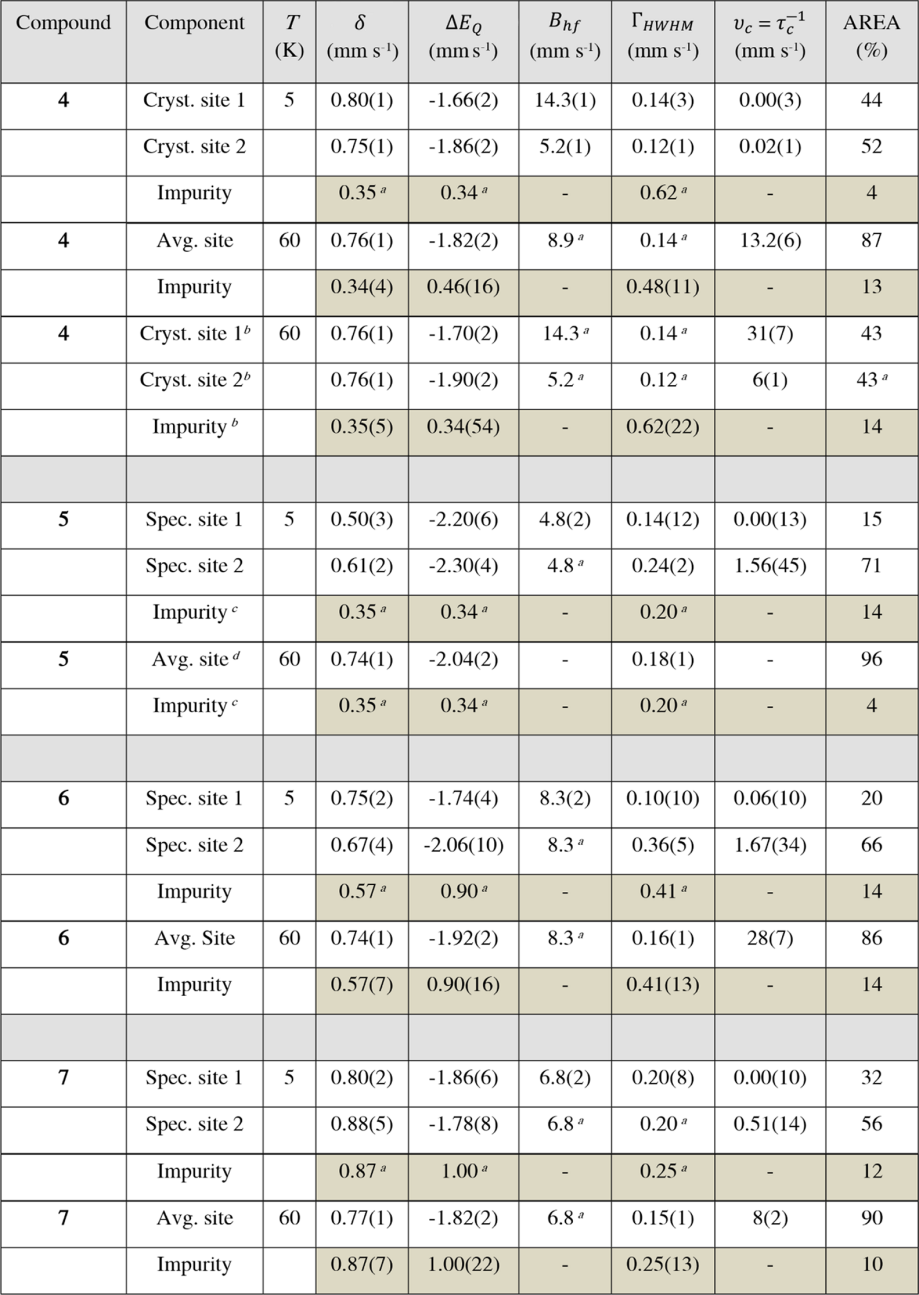
Zero-Field ^57^Fe Mössbauer Parameters for Compounds **4**–**7** ^e^,^f^

a Fixed in the fit.

b Alternative fit with two nonequivalent ^57^Fe sites.

c Parameters (*δ*, Δ*E*_*Q*_) of the impurity contribution were taken from **4**, while the line width was set to the value of the natural line width.

d Here, an integral intensity ratio of A_2_/A_3_ = 1.5(1) was used for the second (A_2_) and third (A_3_) absorption line of the magnetic hyperfine pattern (instead of A_2_/A_3_ = 2, as expected for polycrystalline specimens in zero applied magnetic field), suggesting the presence of texture effects in this sample.^48^ An alternative analysis with a simple doublet of Lorentzian lines revealed identical parameters (cf. Tables S3 and S4) and an intensity ratio of 1.2(1) for the two lines of the doublet, indicating that for **5** the fast dynamic limit is already reached at *T* = 60 K.

e The highlighted rows in the table refer to an unidentified impurity fraction (cf., Figures 5–7 and S9–S13).

f The isomer shift (δ) is specified relative to metallic iron at ambient temperatures and was not corrected for the second-order Doppler shift. Δ*E_Q_* denotes the electric quadrupole splitting, Γ_*HWHM*_ the half (Lorentzian) line width at half maximum and *v*_c_ = $\tau_{c}^{‐1}$ the fluctuation rate of the local magnetic hyperfine field (*B_hf_*) that were determined with the Blume-Tjon relaxation model.^46^ AREA is accounting for the spectral area of a given ^57^Fe site (proportional to the site population).

#### Qualitative Discussion of the Mössbauer Spectra

Interestingly, the absorption spectrum of compound **4** shows a well-resolved magnetic hyperfine pattern at *T* = 5 K (Figure 6), whereas it collapses into an asymmetrically broadened doublet at *T* = 60 K (Figures 5 and S9). DC magnetic-susceptibility measurements rule out the onset of long-range magnetic order (*vide infra*), so the emergence of a static hyperfine field at low temperature must instead reflect a pronounced slowing of the electronic paramagnetic relaxation rate. Furthermore, the low-temperature spectrum of **4** clearly resolves two inequivalent ^57^Fe sites, each with similar isomer shifts (*δ* = 0.80 and 0.75 mm s^–1^, respectively), quadrupole splittings (Δ*E*_*Q*_ = –1.66 and –1.86 mm s^–1^, respectively) and relaxation times, but markedly different magnetic hyperfine fields (*B*_*hf*_ = 14.3 and 5.2 T, respectively) (Table 4). These two components of the Mössbauer spectrum appear in roughly a 1:1 ratio, whereas at *T* = 60 K a (time-averaged) one-component spectrum (beside a small unidentified impurity fraction) is observed. These findings are consistent with the two crystallographically inequivalent iron sites detected by the single crystal X-ray diffraction (see discussion below). For all other investigated compounds (**5**–**7**), the absorption spectrum at *T* = 5 K (Figures 6 and 7) again exhibits two components (subspectra) with similar isomer shift and quadrupole splitting; however (in contrast to the crystallographic site inequivalence observed in compound **4**), now with virtually identical values for the local magnetic hyperfine fields *B*_*hf*_ for both ^57^Fe sites. One subspectrum displays a well-resolved magnetic hyperfine splitting with long relaxation times, while the other remains an asymmetrically broadened doublet akin to the high-temperature line shape, indicating significantly shorter relaxation times. Here, the observation of two spectroscopically nonequivalent ^57^Fe sites is attributed to microstructural inhomogeneities or local disorder rather than true crystallographic inequivalence, leading to a distribution of relaxation times. Qualitatively, the (mean) paramagnetic relaxation time in the polymer **5** is smaller than in the polymer **4**, which may be attributed to a less symmetric coordination sphere in **5** induced by the unsymmetrically substituted amido ligand. This is also in line with our AC magnetic susceptibility studies on **4**–**7** (*vide infra)* and the recently reported results on the Fe­(II) precursors,^16^ which suggest that a more symmetric coordination sphere may promote longer relaxation times. However, for the monomers **6** and **7**, this trend was not observed in the relaxation times determined by zero-field ^57^Fe Mössbauer spectroscopy.

#### Discussion of Two Magnetically Nonequivalent Fe Sites in **4**

In contrast to compounds **5**–**7**, where the occurrence of two dynamically distinct Mössbauer subspectra was attributed to microstructural inhomogeneities, the situation for **4** is more subtle and two alternative explanations can be advanced:

(1) In general, slow paramagnetic (spin–spin) relaxation is characteristic of a large, negative axial zero-field splitting parameter *D*, which at low temperature confines the population to the *m*_*s*_ = ±3/2 Kramers state. Relaxation then proceeds only by Δ*m*_*s*_ = ±1 transitions, slowing the effective spin–spin relaxation. As shown below, the (DC) magnetic susceptibility and the computational studies on **4**–**7** revealed values for the axial ZFS parameter between *D* = –41 and –59 cm^–1^, suggesting that a relevant thermal population of the excited Δ*m*_*s*_ = ±1/2 Kramers state at *T* = 5 K (<4 cm^–1^) can be neglected. Accordingly, a supplementary measurement on **4** at *T* = 10 K (Figure S13) shows virtually no differences in the parameters of the fit (Table S5), when compared with the measurement of **4** at *T* = 5 K. In this regime, the magnitude of the local magnetic hyperfine field (i.e., the contribution of the Fermi contact field) is determined to the first order by the effective *g* value of the given Kramers state. Assuming a perfectly axial *g*-tensor anisotropy (with *g*_*z*_ = 6 and *g*_*x*_ = *g*_*y*_ = 0), a one-component Mössbauer absorption spectrum with characteristic values for the isomer shift, quadrupole splitting and magnetic hyperfine field is expected, contrasting the observation of two magnetically nonequivalent ^57^Fe sites in **4**. Consequently, within this approach, the appearance of a second component in the Mössbauer spectrum with altered magnetic hyperfine field (and only slightly different values for *δ* and Δ*E_Q_*) might suggest a departure from strict axial symmetry, leading to *g* values of *g_z_* < 6.00 and *g*_*y*_ ≈ *g*_*x*_ > 0 and an anisotropic effective hyperfine tensor (e.g., with *A*_*z*_ ≫ *A_y_* ≈ *A*_*x*_ ≠ 0) for the Kramers doublet of the ground electronic state.^49^ However, neither the (DC) magnetic susceptibility measurements nor the computational results support any significant rhombicity in the *g*-tensor for compounds **4**–**7**.
(2) Based on the crystallographic data for **4** the presence of two magnetically nonequivalent iron sites might originate from its crystal structure: the asymmetric unit of **4** contains two crystallographically inequivalent Fe centers. These differ slightly in their Fe–ligand bond lengths and angles (see X-ray crystallography section) and their approximate 1:1 stoichiometric ratio aligns well with the intensity ratio of the two subspectra seen in the Mössbauer spectrum at *T* = 5 K. Nevertheless, to confirm the reproducibility of this behavior and to assess the influence of microstructural or crystallization effects on the relative spectral contributions, an independently prepared sample of **4** (denoted as 4*) was measured at *T* = 10 K (Figure S13). Both compounds exhibit two magnetically inequivalent ^57^Fe sites with essentially identical isomer shifts *δ*, quadrupole splittings Δ*E_Q_*, magnetic hyperfine fields *B_hf_* and fluctuation rates *ν_c_* (cf., Tables S3 and S5). However, their spectral intensity ratios now differ markedly (44:51 in **4** compared to 24:67 in 4*) suggesting that - in addition to the “true” crystallographic inequivalence - minor variations of the crystallization conditions could also affect the microcrystalline structure of individual crystallites in the polycrystalline specimen (i.e., for example, variations in the distribution of repeating units of the polymers or changes in the distribution of particle sizes and grain boundaries for different crystallites in the polycrystalline powder). In this scenario, the number of crystallites with higher microcrystalline disorder might change in the powder (in contrast to the well-ordered and “homogeneous” single crystal), resulting in the observed deviation from a 1:1 ratio.

#### Discussion of Different Magnetic Hyperfine Field Values in Compound **4**

Considering that our magnetic susceptibility measurements and computational results exclude significant rhombicity in the *g*-tensor for compounds **4**–**7**, the two different values for the local magnetic hyperfine fields (*B*_*hf*_) for the two (crystallographically) nonequivalent ^57^Fe sites in **4** might be understood as resulting from a slight canting among the different contributions to the effective magnetic hyperfine field *B*_*hf*_. In zero-field ^57^Fe Mössbauer spectroscopy, *B_hf_* usually arises from the Fermi contact field (*B*_*F*_). However, two additional contributions, the magnetic field (*B*_*L*_) produced by the electronic orbital angular momentum and the magnetic field (*B*_*d*_) due to a nonspherical spin density of the 3d electrons, have also to be considered.^50,51^ Hence, the magnetic hyperfine field *B*_*hf*_ is determined as a vector sum, ${B⃗}_{hf}$ = ${B⃗}_{F}$ + ${B⃗}_{L}$ + ${B⃗}_{d}$ , and depends on the angles enclosed. For compound **4**, assuming an axial-symmetric *g*-tensor anisotropy, the Fermi contact field of *B_F_* = –33 T can be estimated with the “–11 T per unpaired electron” rule.^50,51^ Yet, at *T* = 5 K, the observed magnetic fields for **4** are only *B*_*hf*_ = 14.3 or 5.2 T, implying substantial opposing contributions from *B_L_* and *B*_*d*_. Because the isomer shift and quadrupole splitting of the two ^57^Fe sites in **4** are essentially identical, the difference in *B*_*hf*_ must arise not from unequal magnitudes of *B*_*L*_ and *B_d_* for each ^57^Fe site, but from the angle (*α*) enclosed by *B_L_* + *B*_*d*_ and *B*_*F*_. For example, when the ^57^Fe site with *B*_*hf*_ = 5.2 T is associated with an antiparallel orientation of *B*_*L*_ + *B_d_* relative to *B_F_* (*α* = 180°), a canting of *B*_*L*_ + *B*_*d*_ of ca. 25° (*α* = 180° ± 25°) can be calculated to simulate the observed magnetic hyperfine field of *B*_*hf*_ = 14.3 T for the second ^57^Fe site in **4**. However, it should be stressed at this point, that the given values for *B*_*F*_, *B_L_* + *B*_*d*_ and *α* are only rough estimates; in the absence of further experimental data a more reliable determination of these parameters is not possible without the various simplifying assumptions applied.

### Computational Studies

To gain further insight into the magnetic properties of the iron­(I) complexes, computational studies have been performed on monoanionic model structures of complexes for **4**–**7** (see also Computational Details). DFT/TPSSh/TZVP calculations on the mononuclear iron­(I) computational models (see Figure S14) yielded electron densities ρ at the Fe­(I) nucleus, which were used to compute ^57^Fe isomer shifts δ (Table S6).^52^ The predicted δ values (0.72–0.78 mm s^–1^) agree well with the experimental data (i.e., *δ* = 0.50–0.88 mm s^–1^). Natural population analysis (NPA) of the DFT wave functions (Table S7) partitions charges among the Fe­(I) center, the amido nitrogen donor, and the Cp′ fragment. In all cases, the Fe center carries a +I oxidation state (NPA charge 0.774–0.790) with three unpaired electrons (NPA spin density 2.883–2.896). The amido N charge varies with substituent (SiMe_3_: –1.750/–1.755/–1.772 in **4 (**Fe1)/**4** (Fe1A)/**6**; dipp: –1.293/–1.292 in **5**/**7**), while the Cp′ fragment charge remains essentially constant (−1.218 to –1.208). Valence-shell NPA (Table S8) further reveals partial 3d → 4s charge transfer, giving the Fe 4s orbital an occupancy of 0.394–0.416.

To investigate magnetic anisotropy and ZFS in **4**–**7**, multireference *ab initio* studies on the CAS­(7,10)­SCF/CASPT2/RASSI-SO level of theory were performed. Relative CASSCF and CASPT2 energies (see Tables S9 and S10) show a high-spin (2*S*+1 = 4) ground state for all iron­(I) centers in **4**–**7**. Regarding the CASPT2 energies, the first low-spin (2*S*+1 = 2) state in **4**–**7** shows an energy gap of approximately 12,000 cm^–1^ as compared to the high-spin ground state. The additional inclusion of spin–orbit coupling leads to four Kramers doublets (KDs) below 1,200 cm^–1^ (see Table S11), for which an effective spin Hamiltonian model (*S*_eff_ = 3/2) takes the first two KDs into account. Assuming the first excited KD is the thermal barrier for magnetic relaxation processes, there is a notable difference in these energy barriers [**4** (Fe1): 102 cm^–1^; **4** (Fe1A): 81 cm^–1^; **5**: 97 cm^–1^; **6**: 113 cm^–1^; **7**: 83 cm^–1^]. The corresponding ZFS parametrization (*S*_eff_ = 3/2) is given in Table S12 and exhibits an eas*y*-axis type of magnetic anisotropy (*D* < 0) for the iron­(I) centers in **4**–**7**. Furthermore, only small rhombic ZFS parameters in the range from –0.51 to –0.19 cm^–1^ were calculated, leading to an |*E*/*D*| ratio ≤ 0.01. This is consistent with the nearly linear coordination environment of the iron­(I) centers (Cp'_cent_–Fe–N). The corresponding *g*-factors (*S*_eff_ = 3/2) are also consistent (*g*_*z*_ ≫ *g*_x,y_; *g*_*x*_ ≈ *g*_*y*_) with an eas*y*-axis type of magnetic anisotropy in the ZFS parametrization (*D* < 0; *E* ≈ 0). Moreover, all ground-state KDs in **4**–**7** (*S*_eff_ = 1/2) show a distinct easy axis of magnetization (see Table S13) with *g*_*z*_ values ranging from 8.335 [**4** (Fe1A)] to 8.918 (**6**) and marginal *g*_x,y_ values (*g*_x,y_ ≤ 0.075). The corresponding easy axes of magnetization (*g*_*z*_) for **4**–**7** are depicted in Figure 8 and, as expected, align along the Cp’_cent_–Fe–N vectors.

**Figure 8:**
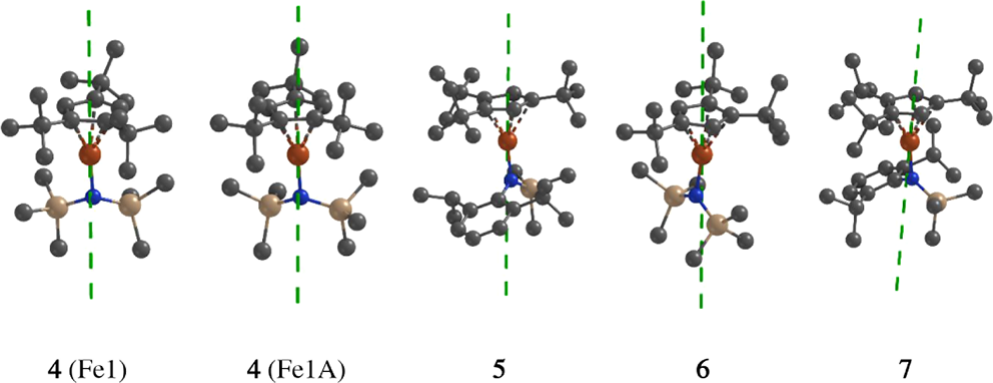
Orientation of the calculated easy axes of magnetization (*g*_*z*_) for the ground state Kramers doublet (*S*_eff_ = 1/2) in the model structures of complexes **4**–**7**. Hydrogen atoms have been omitted for clarity.

### Magnetic Measurements

The magnetic susceptibility of powdered crystalline samples of **4**–**7** was measured in the temperature range 2–200 K with an applied DC field of *H* = 0.2 T (see Figure 9). As expected for mononuclear complexes featuring well-isolated spin centers (also valid for the polymers, see X-ray crystallography section) and substantial magnetic anisotropy, the high temperature values deviate significantly from the spin-only value of 1.875 cm^3^ K mol^–1^ for the *S* = 3/2 ground state. Upon cooling below 50 K, the *χT*_mol_ vs *T* curves exhibit a pronounced decrease, consistent with the depopulation of thermally accessible sublevels due to axial ZFS.

**Figure 9:**
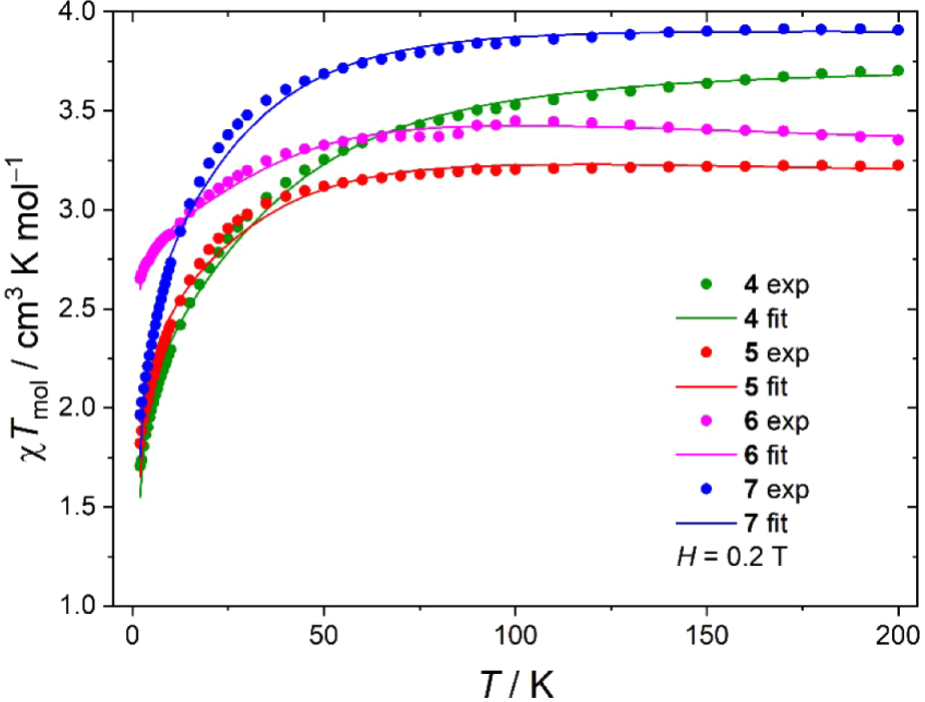
Representation of DC susceptibility recorded at *H* = 0.2 T for complexes **4**–**7** as *χT*_mol_ *vs T* showing experimental data (dots) and fits to eq 1 (lines).

All DC susceptibility data were fitted to the pure spin Hamiltonian of eq 1, incorporating an axial *g*-tensor Zeeman term and an axial ZFS parameter *D*. Neglecting rhombicity is justified by the pseudolinear Fe­(I) coordination sphere and the very small *E*/*D* (≤0.01) values resulting from our *ab initio* calculations.

$$Ĥ=g⃗\mu_{B}BŜ+D[{Ŝ}_{z}^{2}-\frac{1}{3}S(S+1)]$$ 1

As the DC susceptibility is insensitive to *D*, the *D* parameters were fixed to the *ab initio* values for the fit. Furthermore, in complex **4**, which has two crystallographically distinct Fe­(I) sites, the average of the computed *D* values was used in the fit to avoid overparametrization. Hence, only the *g*_∥_ and *g*_⊥_ were fitted, using the *ab initio* values as starting points. Weak intermolecular magnetic coupling was treated via a mean-field approach in the form of a *zJ* term in the PHI program.^53^ The fitted parameters are listed in Table 5, and the resulting curves are shown in Figure 9. The parameters found for compounds **4**–**7** are well within the range observed for other Fe­(I) complexes reported in literature, individual differences can be related to the variation of the ligands coordinating at the Fe­(I) center.^7–10,12–15^

**Table 5:** Parameters Resulting from the Fit of DC Susceptibility Data of Compounds **4–7** to the Hamiltonian in eq 1

	**4**	**5**	**6**	**7**
*g*_∥_	2.919(11)	3.057(11)	3.171(4)	3.243(14)
*g*_⊥_	2.768(12)	2.29(2)	2.274(7)	2.654(18)
*D*/cm^–1^ ^a^	–45.765	–48.45	–59.01	–41.39
*zJ*/cm^–1^	–0.0120(5)	–0.116(4)	–0.0143(11)	–0.120(5)
residual	0.093	0.119	0.026	0.166

a *D* was fixed to the values obtained from *ab initio* calculations.

Magnetization measurements on all four compounds were performed from *T* = 2 to 5 K with magnetic fields up to *H* = 5 T (see Supporting Information, Figure S15), showing no indication of hysteretic behavior. The data were modeled using an *S* = 1/2 pseudospin Hamiltonian featuring only an axial *g-*tensor, which is justified by the exclusive population of the ground state KD at these temperatures. The fitted *g* values, which closely match the *ab initio* results (see Supporting Information, Table S13), are listed in Table S14 and the experimental vs fitted curves are shown in the Supporting Information (Figure S15).

Dynamic AC magnetic susceptibility measurements revealed slow relaxation of magnetization in all four complexes (Table 6). However, for **7** usable AC data were only obtained in the presence of static bias fields (*H*_DC_ = 40 mT; 100 mT). Figure 10 shows the resulting relaxation times τ_C_ (for details see Experimental part and Figures S16–S26 in the SI) as functions of temperature and field. The τ_C_ data were then fitted to the following equation:

**Table 6:** Parameters Resulting from the Fit of Relaxation Times *τ*_C_ as Extracted from AC Magnetic Susceptibility Data of **4**–**7** to eq 2

*H*_DC_	*A*_vib_/s^–1^	*U*_vib_/cm^–1^	*C*/s^–1^
**4**			
0 mT	3.0(7)·10^7^	35.2(9)	249(11)
40 mT	4.6(15)·10^7^	36.8(12)	411(12)
100 mT	1.8(4)·10^7^	35.0(9)	44(12)
**5**			
0 mT	4(4)·10^8^	41(3)	480(40)
40 mT	3.8(6)·10^7^	36.0(6)	37(7)
100 mT	3.5(5)·10^7^	34.8(5)	34(6)
**6**			
0 mT	1.57(9)·10^8^	46.3	3.32(4)·10^3^
40 mT^a^	5.6(13)·10^7^	46.4(9)	-
100 mT^b^	5.4(15)·10^7^	46.2(10)	-
**7**			
40 mT	8.3(16)·10^8^	45.9(7)	-
100 mT	1.7(4)·10^9^	46.0(8)	-

a For **6** at *H*_DC_ = 40 and 100 mT a second exponential term had to be included in the fit fixing *U*_Orb_ to 113 cm^–1^ (energy gap between the first two Kramers doublets from *ab initio* calculational results) and resulting in *A*_Orb_= 2.9(4)·10^13^ s^–1^ (40 mT) and 3.2(5)·10^13^ s^–1^ (100 mT).

b This value was fixed to the average of the two corresponding parameters from the measurements with applied static field, because without static field the data point coverage is poor.

**Figure 10:**
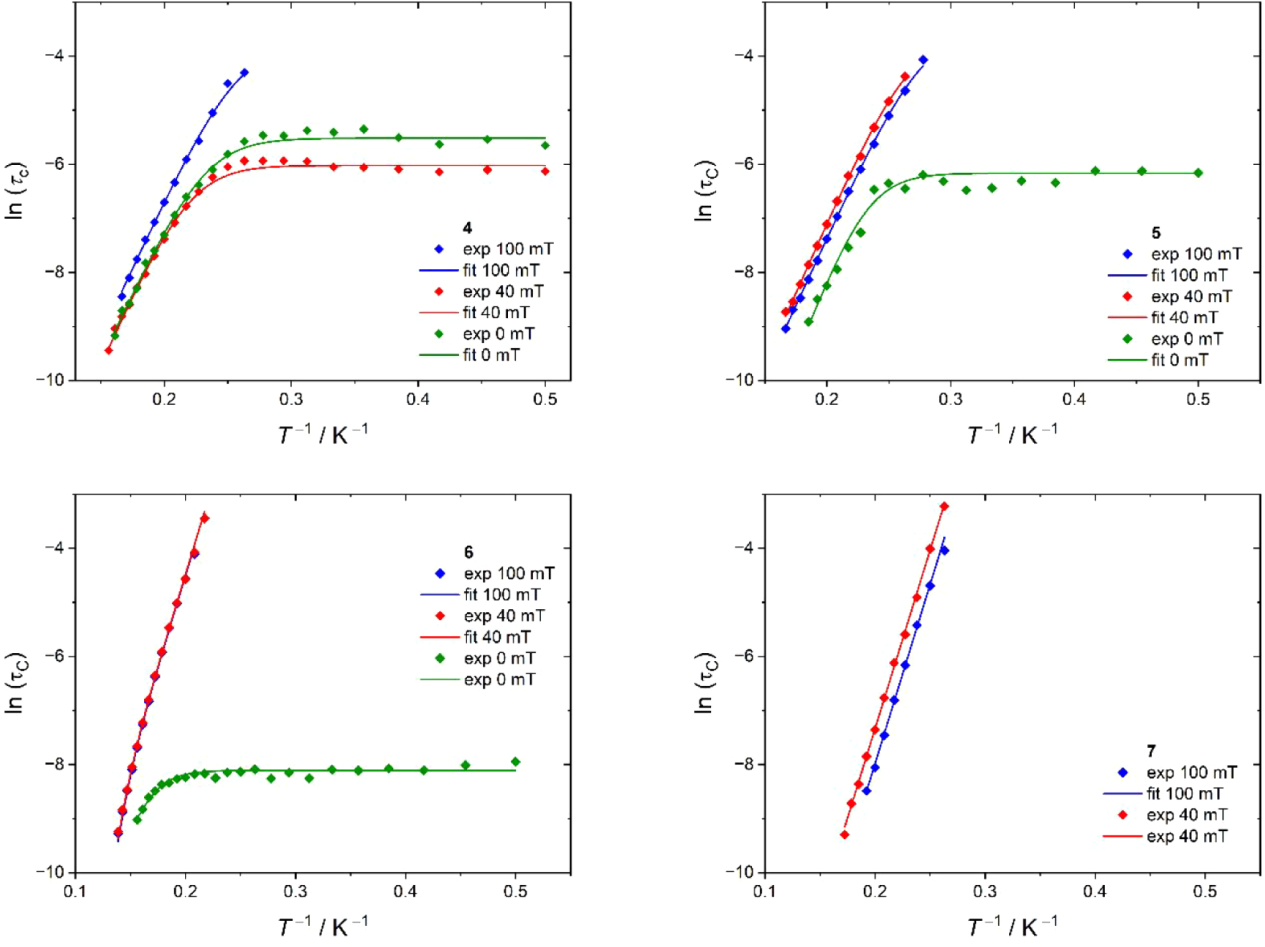
Depiction of the temperature dependence of magnetic relaxation times τ_C_ for compounds **4**–**7** at three different static magnetic fields *H*_DC_ applied. The solid lines represent the fits described in the text with the parameters given in Table 5.

2 $$\tau_{C}^{-1}=A_{\mathrm{Orb}}\cdot \exp\left(\frac{-U_{\mathrm{Orb}}}{k_{B}T}\right)+A_{\mathrm{vib}}\cdot \exp\left(\frac{-U_{\mathrm{vib}}}{k_{B}T}\right)+C$$

where *U*_vib_ represents a vibration-assisted relaxation channel, *U*_Orb_ the thermal barrier via the first excited Kramers doublet, and *C* the quantum-tunneling contribution (QTM).

The resulting parameters are provided in Table 6 and the corresponding fits are depicted in Figure 8. At least one relaxation process of exponential type (*A*_vib_, *U*_vib_) can be observed in each complex, and the extracted barriers *U*_vib_ do not coincide with the energy gaps to the first excited Kramers doublet obtained from the *ab initio* calculations. This unambiguously identifies a vibrationally assisted relaxation pathway being present.^31–33,54^ Notably, the polymeric complexes **4** and **5** exhibit *U*_vib_ ≈ 35 cm^–1^, whereas the monomeric compounds **6** and **7** show *U*_vib_ ≈ 46 cm^–1^. Because of these very similar values, we assume that comparable - most likely low-energy intramolecular - vibrational modes are responsible for this relaxation path. The exponential term containing *U*_vib_ thus reflects the activation of specific phonon modes that efficiently couple to the spin manifold. We therefore attribute the dominant vibrational contribution to a mode involving the Fe­(I) center and the Cp’ ligand: its bridging coordination in **4** and **5** lowers the vibration energy relative to the terminal Cp’ in **6** and **7**, explaining the barrier difference. However, a conclusive assignment of the specific normal modes responsible is a complex task - especially for Fe­(I) systems, where precedent is lacking - and lies beyond the scope of this work.^33,55^

In the absence of static magnetic fields significant QTM is observed for all compounds except **7**, for which the QTM rate at zero field is simply too fast to be detected within the relaxation time window feasible within standard AC susceptibility measurements. For **6** the small static fields of 40 and 100 mT effectively suppress QTM, which is the expected trend for mononuclear uncoupled systems. In contrast, for **5** the magnetic field-induced QTM suppression appears less efficient. A somewhat unexpected field dependence of QTM is observed in **4**: the QTM rate at 40 mT is significantly increased in comparison to zero field, while at *H*_DC_ = 100 mT it is almost entirely suppressed. Such phenomena are well-known, e.g., for dimeric lanthanide species featuring weak antiferromagnetic exchange,^56–58^ and are referred to as exchange bias. Comparable effects are reported for 3d metal complexes by Christou et al.,^59,60^ but are yet a rare observation, because in classical bridged structure motifs the magnetic interaction is much stronger as compared to 4f metal complexes. In here, the weak intermolecular interaction (formerly parametrized as *zJ*) might be responsible for this peculiar field dependence of QTM.

Compound **5** exhibits an additional exponential-type relaxation process within the available measurement range, with an energy barrier close to the 2*D* value determined by *ab initio* calculations and consequently fixed at this value. Therefore, this process can be rationalized as relaxation over the thermal spin-reversal barrier. However, the parameters of this process are still not well determined by the available data points, and the result should therefore be interpreted with appropriate caution.

In principle, Mössbauer spectroscopy can provide information concerning magnetization dynamics which are not readily accessible by AC magnetic susceptibility studies. This concept was previously demonstrated on mononuclear Fe­(I) complexes by Long et al.^8^ In this approach, the fluctuation rate of the local magnetic hyperfine field, determined within the Dattagupta and Blume^61^ model, was associated with the electronic magnetic relaxation time (τ_C_), as obtained by AC magnetic susceptibility. Unfortunately, it is difficult to directly apply this very elegant approach to our set of Fe­(I) complexes **4**–**7**. As discussed above, the Mössbauer spectra for **4**–**7** indicate a distribution of local relaxation times (which is clearly visible at low temperatures), and additionally as illustrated for compound **4**, the relative ratios of the different contributors may vary from sample to sample (even when the sample was prepared by an identical synthetic protocol (Table S5). This limits the number of reliable fluctuation rates extractable from our zero-field Mössbauer experiments and, hence, we only use the values at *T* = 60 K (or, respectively, at *T* = 20 K for **5**) (Table 4) to improve the precision of the vibrationally assisted relaxation parameters (*U*_vib_ and *A*_vib_) obtained from our AC magnetic susceptibility measurements without an applied static magnetic field (Figure S27). These combined analyses indicate that the barriers *U*_*vib*_ for all four compounds converge to nearly the same value (Table S15), implying that a common Cp′-centered vibrational mode governs this relaxation process. Furthermore, a direct comparison with the Fe­(I) complex studied by Long et al. is not possible because they modeled this process with an outdated power-law term,^8^ but it is noteworthy that their reported energy gap to the first excited Kramers doublet is roughly twice that of our compounds **4**–**7**.

## Conclusions

We succeeded in the reduction of the iron­(II) half-sandwich complexes [Cp’FeN­(R)­(SiMe_3_)] (*R* = SiMe_3_ (**2**), dipp (**3**)) with KC_8_ forming the polymeric complexes {[Cp’Fe­(N­(SiMe_3_)_2_)]­K}_n_ (**4**) and {[Cp’Fe­(N­(dipp)­(SiMe_3_))]­K}_n_ (**5**). In the presence of 18-crown-6 these polymers can be broken up and the monomeric iron­(I) compounds [Cp’Fe­(N­(SiMe_3_)_2_)]­[K­(18-crown-6)] (**6**) and [Cp’Fe­(N­(dipp)­(SiMe_3_))]­[K­(18-crown-6)] (**7**) were successfully isolated and structurally characterized. All of these complexes exhibit slow paramagnetic relaxation as evidenced by their zero-field ^57^Fe Mössbauer spectra. *Ab initio* calculations on complexes **4**–**7** revealed an *S* = 3/2 ground-state Kramers doublet, that lies ca. 100 cm^–1^ below the first excited state, and the computations are in good agreement with the experimental Mössbauer parameters and *g*-tensors. By fixing these calculated values in our spin-Hamiltonian model, we avoid overparameterization and achieve excellent fits of the DC magnetic data *via* full matrix diagonalization. AC susceptibility measurements then reveal zero-field slow relaxation of magnetization for **4**–**6** and field-induced slow relaxation for **7**. Notably, complex **4** exhibits an unusual field dependence of quantum tunneling of magnetization akin to the exchange-bias behavior seen in lanthanide dimers. Throughout all four compounds the accessible magnetic relaxation range at elevated temperatures is governed by a vibrationally assisted process, which is presumably related to the unsymmetric Cp’ moiety. Thus, using *C*_5_ symmetric Cp derivatives for similar syntheses will be the target of future works to widen this bottleneck of magnetic relaxation and improve the slow relaxation of magnetization in those compounds.

## Experimental Section

### General Considerations

All manipulations were performed under N_2_ in a MBraun Unilab glovebox or by standard Schlenk techniques. Solvents were dried over Na/benzophenone, then distilled and degassed before use. [Cp’Fe­{N­(SiMe_3_)_2_}] (**2**),^34^ [Cp’Fe­{N­(dipp)­(SiMe_3_)}] (**3**),^16^ and KC_8_ ^62^ were synthesized according to literature procedures. All other commercial reagents were used as received unless otherwise stated. NMR spectra were recorded at 25 °C on Bruker AVII300 (300 MHz), Bruker AVIII400 (400 MHz) and Bruker AVIIIHD500 (500 MHz) spectrometers. ^1^H chemical NMR shifts (δ, ppm) were referenced to residual solvent proton signals, and half-height line widths (ν_1/2_) are given in Hertz (Hz). Elemental analyses were carried out on the Vario-Micro-Cube-System. Melting points were determined visually with an MPM-HV2 apparatus and are uncorrected. Single crystals for X-ray diffraction analysis were mounted in inert oil on glass fibers or nylon loops and measured on Oxford Diffraction systems using mirror-focused Cu Kα or monochromatic Mo Kα radiation. Structures were refined anisotropically against *F* ^2^ with SHELXL-97,^63,64^ placing H atoms in riding positions or as rigid methyl groups.

### Synthesis of {[Cp’Fe­(N­(SiMe_3_)_2_)]­K}_n_(4)

[Cp’Fe­{N­(SiMe_3_)_2_}] (**2**) (100 mg, 0.222 mmol, 1.0 equiv) was dissolved in Et_2_O (5 mL), and a suspension of KC_8_ (33 mg, 0.245 mmol, 1.1 equiv) in Et_2_O (4 mL) was added dropwise with stirring, provoking immediate formation of a black precipitate. After 15 min at room temperature, the mixture was filtered through Celite and the filtrate concentrated under reduced pressure to afford a light-brown residue. Recrystallization was achieved by layering the Et_2_O solution with *n*-hexane and cooling to –30 °C, yielding dark-brown crystals that were collected and dried in vacuo for 15 min. Alternatively, if the concentrated filtrate remained oily, addition of *n*-hexane (1–2 mL) and brief stirring induced precipitation of an olive-green solid. The supernatant was decanted, and the solid was dried under vacuum for 15 min to afford the product as a green-brownish material. Yield: 96 mg (0.196 mmol, 88%). Decomp. point: 85 °C. Anal. Calc. (%) for C_23_H_47_NSi_2_KFe (488.75 g/mol) C 56.52, H 9.69, N 2.87; found C 55.72, H 9.43, N 2.40. ^1^H NMR (300 MHz, THF-*d*_8_): δ = 0.0 (ν_1/2_ = 450 Hz, 9 H, Si­(CH_3_)_3_), −0.4 (ν_1/2_ = 330 Hz, 9 H, C­(CH_3_)_3_), −13.1 (ν_1/2_ = 860 Hz, 18 H, 2 × C­(CH_3_)_3_) ppm. The resonance for the Cp-bound protons was not observed.

### Synthesis of [Cp’Fe­(N­(SiMe_3_)_2_)]­[K­(18-crown-6)­(thf)_2_] (**6**)

[Cp’Fe­{N­(SiMe_3_)_2_}] (**2**) (100 mg, 0.222 mmol, 1.00 equiv) and 18-crown-6 (56 mg, 0.211 mmol, 0.95 equiv) were dissolved in Et_2_O (5 mL) and stirred for 5 min. A suspension of KC_8_ (33 mg, 0.245 mmol, 1.10 equiv) in Et_2_O (4 mL) was then added, immediately producing a deep red-brown solution and a black precipitate. After 15 min at room temperature, the reaction mixture was filtered through Celite and the filtrate concentrated under reduced pressure to afford a brown solid. Recrystallization from THF layered with *n*-hexane at −30 °C yielded dark-brown crystals, which were dried in vacuo for 15 min. Alternatively, compound **6** may be prepared by stirring a THF solution of **4** (1.00 equiv) and 18-crown-6 (1.00 equiv) for 2 h, then recrystallizing from THF/*n*-hexane at −30 °C under identical layering and drying conditions. Prolonged exposure to dynamic vacuum removes the coordinated THF at the cation [K­(18-crown-6)­(thf)_2_]^+^. Yield: 141 mg (0.187 mmol, 84%). Decomp. point: 65 °C. Anal. Calc. (%) for C_35_H_71_NO_6_Si_2_KFe (753.07 g/mol) C 55.82, H 9.50, N 1.86; found C 55.51, H 9.46, N 1.62. ^1^H NMR (300 MHz, THF-*d*_8_): δ = 2.4 (ν_1/2_ = 1500 Hz, 24 H, 12 × CH_2_ (18-crown-6)),), 0.1 (ν_1/2_ = 90 Hz, 9 H, Si­(CH_3_)_3_), −2.9 (ν_1/2_ = 500 Hz, 9 H, C­(CH_3_)_3_), −12.0 (ν_1/2_ = 450 Hz, 18 H, 2 × C­(CH_3_)_3_) ppm. The resonance for the Cp-bound protons was not observed.

### Synthesis of {[Cp’Fe­(N­(dipp)­(SiMe_3_))]­K}_n_(5)

[Cp’Fe­{N­(dipp)­(SiMe_3_)}] (**3**) (50 mg, 0.0930 mmol, 1.0 equiv) was dissolved in Et_2_O (5 mL). A suspension of KC_8_ (14 mg, 0.102 mmol, 1.1 equiv) in Et_2_O (4 mL) was added dropwise with stirring, immediately yielding a brown solution and a fine black precipitate. After 15 min at 20 °C, the mixture was filtered through Celite and the filtrate concentrated under vacuum to afford a red-brown solid. Slow cooling of a fresh Et_2_O solution at –30 °C furnished red-brown crystals, which were collected and dried *in vacuo* for 15 min. Yield: 35 mg (0.0607 mmol, 65%). Decomp. point: 72 °C. Anal. Calc. **(%)** for C_32_H_55_NSiKFe (576.83 g/mol) C 66.63, H 9.61, N 2.43; found C 64.27, H 8.99, N 1.49. ^1^H NMR (300 MHz, THF-*d*_8_): δ = 31.6 (ν_1/2_ = 1200 Hz, 2 H, Cp-H), 18.3 (ν_1/2_ = 200 Hz, 1 H, ^i^Pr-CH), 16.7 (ν_1/2_ = 200 Hz, 6 H, ^i^Pr-CH_3_), 1.1 (ν_1/2_ = 220 Hz, 9 H, Si­(CH_3_)_3_), −5.1 (ν_1/2_ = 300 MHz, 9 H, C­(CH_3_)_3_), −11.6 (ν_1/2_ = 320 Hz, 18 H, 2 × C­(CH_3_)_3_), −25.3 (ν_1/2_ = 250 MHz, 6 H, ^i^Pr-CH_3_) ppm.

### Synthesis of [Cp’Fe­(N­(dipp)­(SiMe_3_))]­[K­(18-crown-6)­(thf)_2_] (7)

[Cp’Fe­{N­(dipp)­(SiMe_3_)}] (**3**) (118 mg, 0.219 mmol, 1.00 equiv) and 18-crown-6 (55 mg, 0.208 mmol, 0.95 equiv) were dissolved in THF (15 mL) and stirred for 5 min. A suspension of KC_8_ (36 mg, 0.263 mmol, 1.20 equiv) in THF (4 mL) was then added under stirring, immediately giving a deep red-brown solution and a fine black precipitate. After stirring for 15 min at ambient temperature, the mixture was filtered through Celite and the combined filtrate concentrated *in vacuo* to yield a brown oily residue. This residue was washed with *n*-hexane (ca. 10 mL) until the washing solutions remained colorless, then dried under reduced pressure for 15 min to afford the product as a red-brown powder. Single crystals suitable for X-ray diffraction were grown by slow diffusion of *n*-pentane into a saturated THF solution of the product at −30 °C. Alternatively, complex **7** may also be prepared by stirring a THF solution of **5** (1.00 equiv) and 18-crown-6 (1.00 equiv) for 2 h, followed by recrystallization from THF layered with *n*-hexane or *n*-pentane at −30 °C. Prolonged exposure to dynamic vacuum removes the coordinated THF at the cation [K­(18-crown-6)­(thf)_2_]^+^. Yield: 87 mg (0.103 mmol, 47%). Decomp. point: 120 °C. Anal. Calc. (%) for C_44_H_79_NO_6_SiKFe (841.15 g/mol) C 62.83, H 9.47, N 1.67; found C 62.14, H 9.24, N 1.65. ^1^H NMR (300 MHz, THF-*d*_8_): δ = 18.2 (ν_1/2_ = 150 Hz, 1 H, ^i^Pr-CH), 16.8 (ν_1/2_ = 160 Hz, 6 H, ^i^Pr-CH_3_), 3.8 (ν_1/2_ = 50 Hz, 24 H, 12 × CH_2_ (18-crown-6)), 0.1 (ν_1/2_ = 100 Hz, 9 H, Si­(CH_3_)_3_), −4.9 (ν_1/2_ = 300 Hz, 9 H, C­(CH_3_)_3_), −11.4 (ν_1/2_ = 300 Hz, 18 H, 2 × C­(CH_3_)_3_), −25.6 (ν_1/2_ = 220 Hz, 6 H, ^i^Pr-CH_3_) ppm. The resonances for the Cp-bound protons and the aromatic phenyl protons were not observed.

### Zero-Field ^57^Fe Mössbauer Spectroscopy

Polycrystalline powders of compounds **4–7** were prepared with an area density corresponding to ca. 0.21 (**4**), 0.15 (4*), 0.14 (**5**), 0.14 (**6**) and 0.08 mg ^57^Fe cm^–2^ (**7**) and filled in sample containers made of PEEK (polyether ether ketone) or Teflon. The measurements were performed on a commercial (*WissEl* and *Halder*) transmission spectrometer with sinusoidal velocity sweep. The velocity calibration was done with an α-Fe foil at ambient temperature. The minimum experimental line widths (fwhm) were < 0.22 mm s^–1^.

The temperature-dependent measurements were executed with a (*CryoVac*) continuous-flow cryostat with helium exchange gas adjusted at a pressure of ca. 10–50 mbar during the measurements. The temperature was controlled and recorded with a calibrated Si diode, located close to the sample container, providing a temperature stability of better than ±0.1 K. The nominal activity of the Mössbauer source was 50 mCi of ^57^Co in a rhodium matrix, which was stored at ambient temperatures during the measurements. The isomer shifts were specified relative to metallic iron at room temperature but were not corrected in terms of the second-order Doppler shift. The data analyses were carried out on basis of the Blume-Tjon relaxation model,^46^ using the software packages Recoil^65^ and Mathematica.^66^

### Computational Details

DFT computational studies for **4**–**7** were performed with the Turbomole 7.6 package of programs, for which atomic positions of the anionic Fe­(I) complex fragments were taken from the corresponding X-ray crystallographic structures. The resulting five monoanionic Fe­(I) model structures (**4** (Fe1), **4** (Fe1A), **5**-**7**) used for the calculations are depicted in Figure S16. The positions of all hydrogen atoms were optimized at RI-DFT-D3 ^67–71^ /PBE0 ^72,73^ /TZVPP^74^ level of theory. Subsequently, single-point DFT/TPSSh^75,76^ /TZVP^77^ calculations have been performed to obtain the electron density ρ at the iron nucleus position to calculate the ^57^Fe Mössbauer isomer shift δ^52^ (see Table S6) and a natural population analysis (NPA) of the valence shell of the Fe­(I) centers (see Tables S7 and S8).

Multireference *ab initio* calculations based on CAS­(7,10)­SCF/CASPT2 were performed with the OpenMolcas package of programs in version 18.09 to calculate the energy spectrum of the spin–orbit coupled states as well as the corresponding magnetic parameters.^78^ For all *ab initio* calculations ANO-RCC basis sets^79,80^ (Fe and donor atoms: ANO-RCC-VTZP; remaining atoms: ANO-RCC-VDZ) have been employed in combination with a scalar-relativistic second-order Douglas–Kroll–Hess Hamiltonian. The Cholesky decomposition of integrals was used as implemented in OpenMolcas to speed up calculations. State-average CASSCF calculations (for energies see Table S9) were performed with 7 electrons in 10 orbitals (3d and 4d shell) for 10 quartet (^4^F and ^4^P) and 40 doublet states (^2^G, ^2^P, ^2^H, ^2^D, ^2^D, ^2^F). Additional dynamic correlation was subsequently treated by CASPT2 (see Table S10) based on the optimized SA-CASSCF wave function for all quartets and the lowest 12 lowest doublet states. CASSCF/CASPT2/RASSI-SO calculations were carried out to take spin–orbit coupling adequately into account (see Table S11), which allows mixing of different multiplicities. Magnetic properties such as ZFS parameters and *g* values have been obtained by the SINGLE_ANISO module of OpenMolcas.

### Magnetic Measurements

Magnetic susceptibility data were acquired on polycrystalline samples under rigorously inert conditions. Each sample was precooled on dry ice, transferred directly into a glovebox, and loaded into a custom-made, hermetically sealed sample holder, in which it was also coarsely ground. Then, insertion into the cold MPMS-5 SQUID equipped with a 5 T magnet was carried out as fast as possible; the transfer to the machine again utilized an additional Schlenk vessel. Sample masses were determined by weighing the loaded holder immediately after the measurement and subtracting the known tare weight. DC susceptibility data were collected over the 2–200 K range. AC susceptibility was measured between 2 and 15 K at excitation frequencies from 10 to 1500 Hz and under static fields of 0, 40, and 100 mT. In zero field, all compounds except **7** exhibited well-resolved out-of-phase (χ″) signals. Finally, isothermal DC magnetization was recorded from 2 to 5 K in fields up to 5 T. Without magnetic field significant and processable imaginary contributions to the AC susceptibility were observed for all samples, except **7**. The relaxation times τ_c_ were extracted from a fit of the isothermal AC susceptibility data according to

$$\chi \left(\omega \right)=\chi_{S}+\frac{\chi_{0}-\chi_{S}}{1+{\left(i\omega \tau_{c}\right)}^{1-\alpha }}$$

The DC data were corrected for diamagnetic contributions of the sample and the sample holder. Processing of the data was performed with Origin2024 ^81^ and fittings of the DC magnetic data performed with PHI^53^ program.

## Supplementary Material



## Notes

The following files are available free of charge. NMR spectra, crystallographic data, additional zero-field ^57^Fe Mössbauer spectra, computational details, Mössbauer extended magnetic relaxation (PDF)
