# Supplementary material for: A Combined Magnetic and Mössbauer Spectroscopic Study of High-Spin Half-Sandwich Fe(I) Complexes in Monomeric and Polymeric Configurations
Source: Inorg Chem. 2025 Nov 18;64(48):23487–502. doi: 10.1021/acs.inorgchem.5c03729 (PMC12690587; doi:10.1021/acs.inorgchem.5c03729)
Supplement: Supplementary file 1 [file ic5c03729_si_001.pdf]

Supporting Information

A combined magnetic and Mössbauer spectroscopic study of high-spin half-sandwich Fe(I) complexes in monomeric and polymeric configurations

Katharina Münster,[a] Dirk Baabe,[a] Jan Raeder,[a] Benjamin Kintzel,[b] Oluseun Akintola, [b] Michael Böhme,[b] Winfried Plass,[b,] and Marc D. Walter[a,]**

[a] Institut für Anorganische und Analytische Chemie, Technische Universität Braunschweig, Hagenring 30, 38106 Braunschweig, Germany

[b] Institut für Anorganische und Analytische Chemie, Friedrich-Schiller-Universität Jena, Humboldtstraße 8, 07743 Jena, Germany

*Corresponding authors. E-mail: sekr.plass@uni-jena.de, mwalter@tu-bs.de

Table of Contents

1. NMR Spectroscopy **S2**
2. X-ray Crystallography **S5**
3. Zero-field ^{57}Fe Mössbauer Spectroscopy **S9**
4. Computational Details **S13**
5. Magnetic Measurements **S18**
6. Mössbauer extended Magnetic Relaxation **S30**
7. References **S30**

1. NMR Spectroscopy

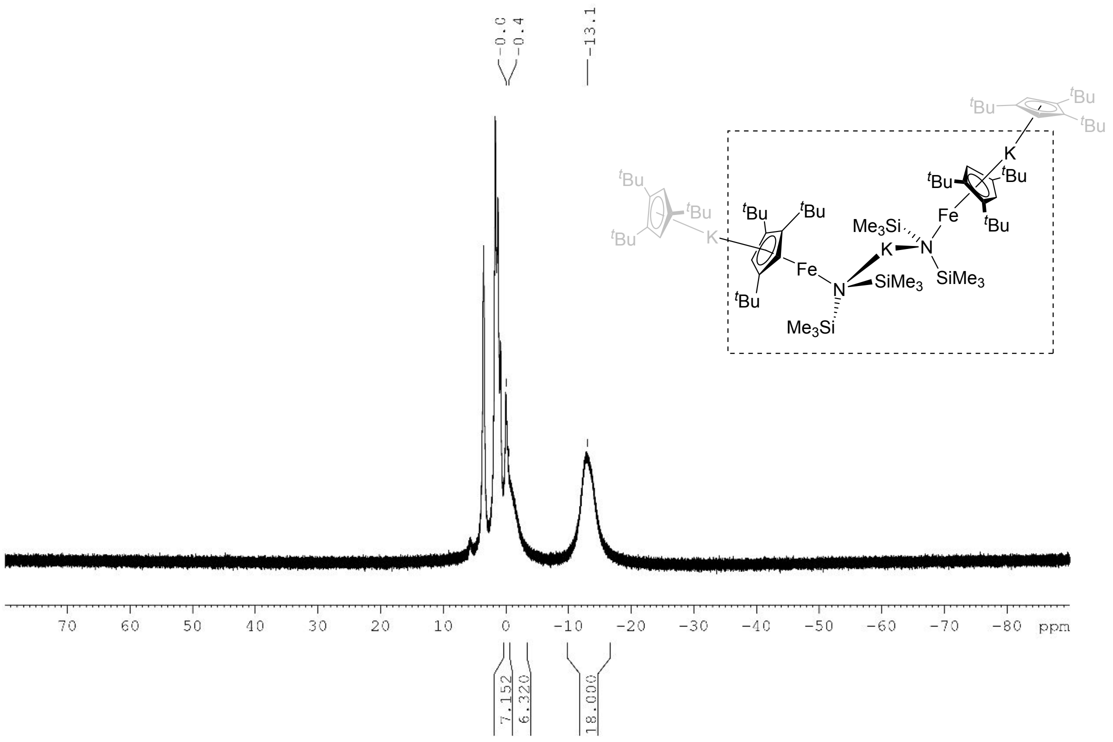

Figure S1. ^{1}H NMR spectrum (300 MHz, THF-d_8) of **4**.

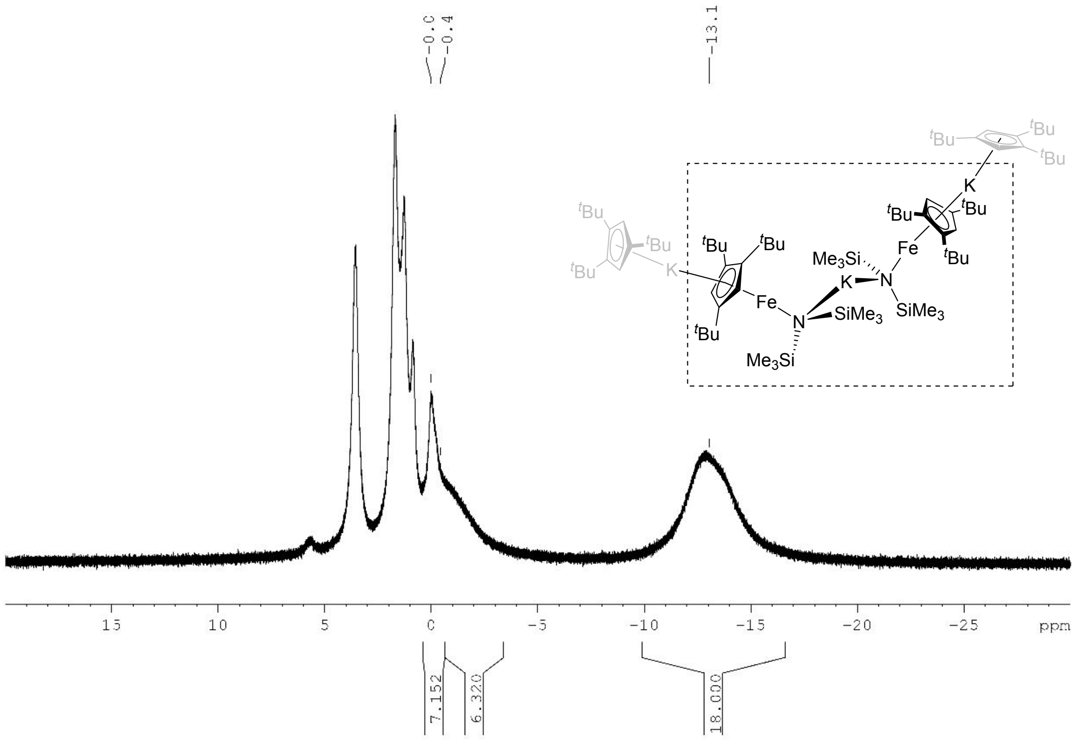

Figure S2. Expansion from the ^{1}H NMR spectrum (300 MHz, THF-d_8) of **4**. The marked peaks correspond to the product, but because of overlap with n-hexane (residual solvent from the reaction) and the residual proton signals of THF-d_8, exact integration was not feasible.

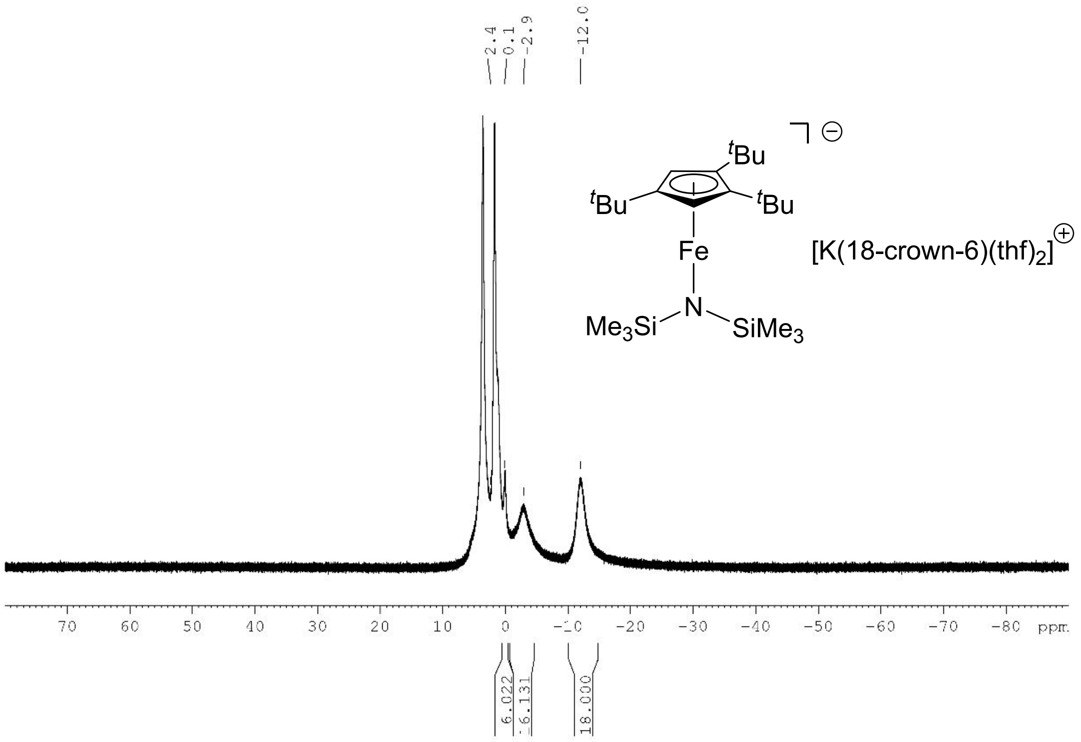

Figure S3. ^{1}H NMR spectrum (300 MHz, THF-d_8) of **6**.

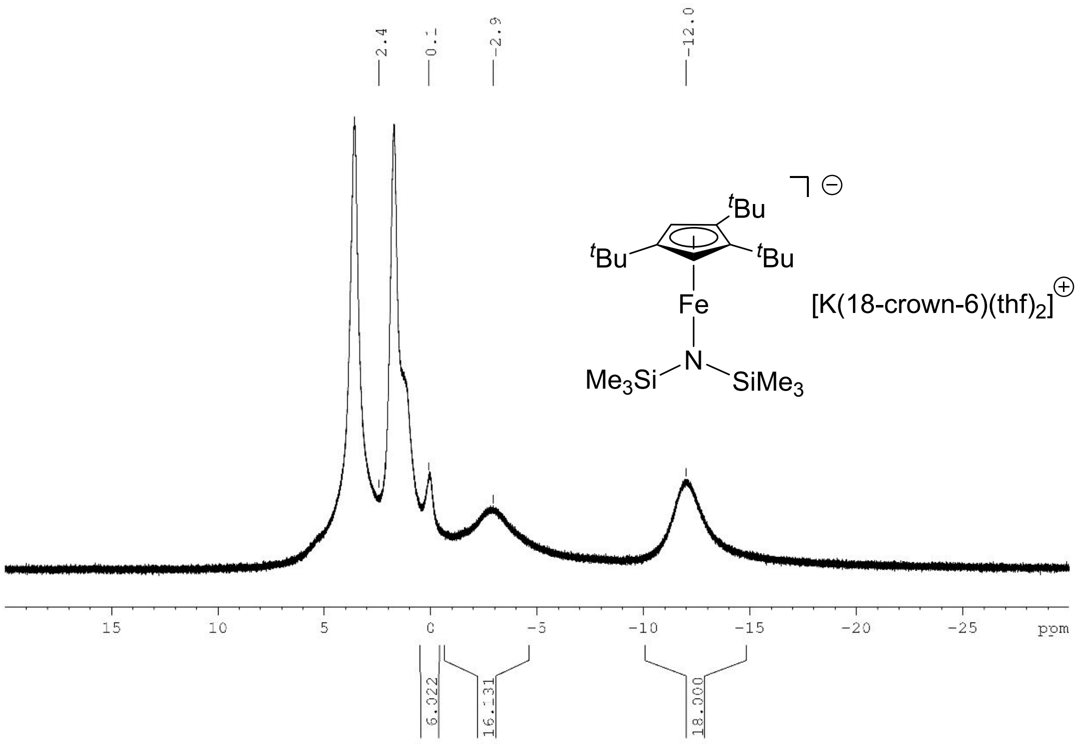

Figure S4. Expansion from the ^{1}H NMR spectrum (300 MHz, THF-d_8) of **6**. The marked peaks correspond to the product, but because of overlap with n-hexane (residual solvent from the reaction) and the residual proton signals of THF-d_8, exact integration was not feasible.

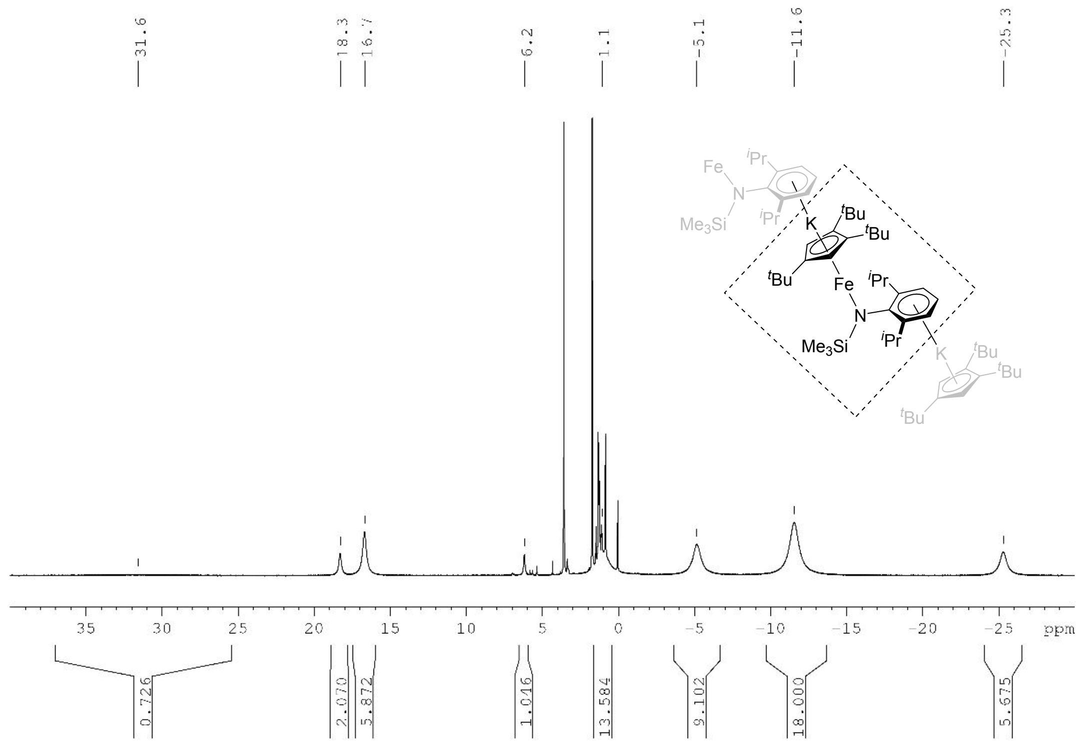

Figure S5. ^1H NMR spectrum (300 MHz, THF-d_8) of **5**. The marked peaks correspond to the product. The solution contains residual n-hexane from the reaction.

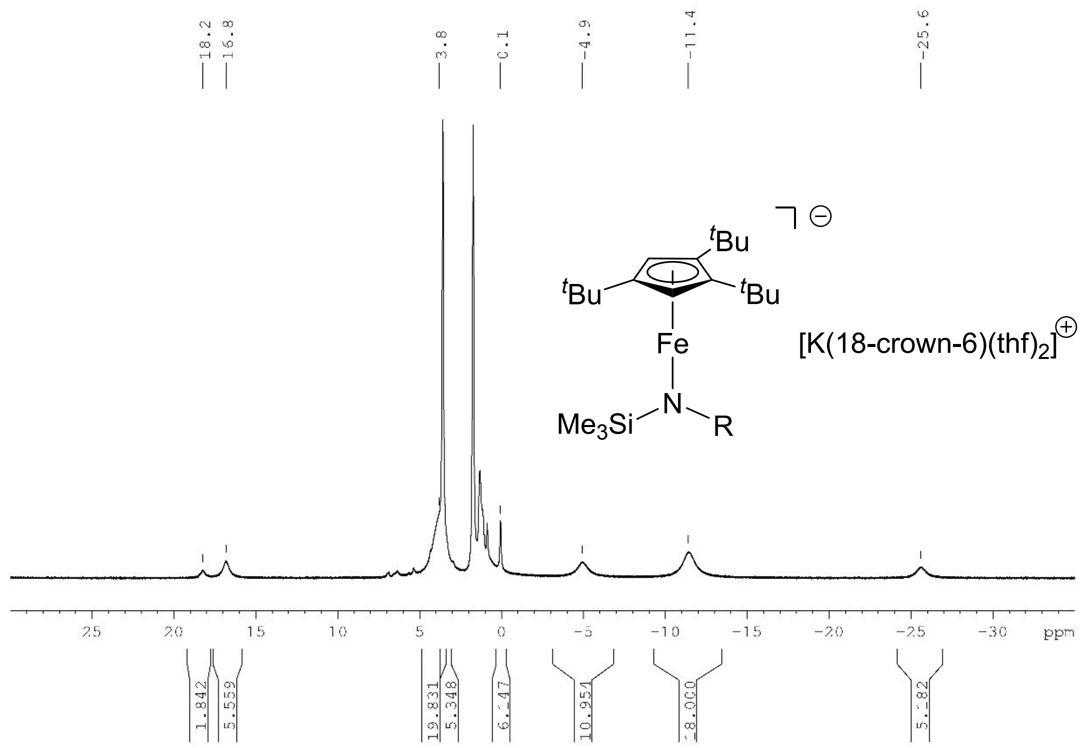

Figure S6. ^1H NMR spectrum (300 MHz, THF-d_8) of **7**. The marked peaks correspond to the product. The solution contains residual n-hexane from the reaction.

2. X-ray Crystallographic Details

2.1 Refinement special details

2.1.1. Complex 4

All H atoms were placed in idealized positions and refined using a riding model, with a common U restrained to be 1.2 (1.5 for methyl groups) times the equivalent isotropic displacement parameter of the parent atom. Ordered methyl groups were treated as rigid bodies and allowed to rotate around the E-CH_3 bond. The structure was found to be polymeric.

2.1.2. Complex 6 (T = 100 K)

All H atoms were placed in idealized positions and refined using a riding model, with a common U restrained to be 1.2 (1.5 for methyl groups) times the equivalent isotropic displacement parameter of the parent atom. Methyl groups were treated as rigid bodies and allowed to rotate about the E-CH_3 bond but not tip. The disordered THF molecules coordinated to the potassium atoms K1 and K2 were modelled using EADP, DANG, DFIX and ISOR. Three missing FCF reflections between θ_{min} and $\sin(\theta)/\lambda = 0.600$.

2.1.3. Complex 6 (T = 80 K)

Refined as a 2-component twin. The twin factor refined to 0.168(2). A disordered tert-butyl group and a THF molecule were modelled using parts 1, 2 and 3, 4, respectively, employing EADP constraints. All H atoms were placed in idealized positions and refined using a riding model, with a common U restrained to be 1.2 (1.5 for methyl groups) times the equivalent isotropic displacement parameter of the parent atom. Methyl groups were treated as rigid bodies and allowed to rotate about the E-CH_3 bond but not tip. The data were acquired at T = 80 K.

2.1.4. Complex 5

All H atoms were placed in idealized positions and refined using a riding model, with a common U restrained to be 1.2 (1.5 for methyl groups) times the equivalent isotropic displacement parameter of the parent atom. Methyl groups were treated as rigid bodies and allowed to rotate about the E-CH_3 bond but not tip.

2.1.5. Complex 7

All H atoms were placed in idealized positions and refined using a riding model, with a common U restrained to be 1.2 (1.5 for methyl groups) times the equivalent isotropic displacement parameter of the parent atom. Ordered methyl groups were treated as rigid bodies and allowed to rotate around the E-CH_3 bond. Two *tert*-butyl groups and two thf ligands were found disordered and refined with a disorder model comprising two different occupations.

Table S1. Solid-state X-ray diffraction data for complexes 4 - 7.

	**4**	**5**	**6 (100 K)**	**6 (80 K)**	**7**
**CCDC**	2479390	2478448	2478449	2478450	2479389
**Chemical formula**	$C_{46}H_{94}N_2Si_4K_2Fe_2$	$C_{32}H_{55}NSiKFe$	$C_{43}H_{87}NO_8Si_2KFe$	$C_{43}H_{87}NO_8Si_2KFe$	$C_{52}H_{95}NO_8SiKFe$
**Formula mass / g/mol**	977.49	576.81	897.26	897.26	985.32
**Crystal system**	orthorhombic	triclinic	triclinic	triclinic	triclinic
***a*/Å**	11.86150(10)	9.8282(2)	9.5770(2)	9.8298(5)	10.85311(8)
***b*/Å**	18.4415(2)	10.3313(4)	9.85550(10)	9.9333(4)	13.19456(7)
***c*/Å**	25.9788(3)	18.6471(4)	27.25620(10)	26.5833(11)	21.49825(10)
**α/°**	90	87.919(2)	89.6650(10)	86.054(3)	102.5596(4)
**β/°**	90	78.154(2)	86.6050(10)	82.277(4)	101.2575(5)
**γ/°**	90	64.051(4)	89.8130(10)	87.346(4)	101.8009(5)
**Unit cell volume/Å^3**	5682.70(10)	1663.17(10)	2568.04(6)	2564.2(2)	2847.35(3)
**Temperature/K**	100.00(10)	100.00(10)	100.00(10)	80.0(6)	105(8)
**Space group**	$P2_12_12_1$	$P\overline{1}$	$P\overline{1}$	$P\overline{1}$	$P\overline{1}$
**No. of formula units per unit cell, *Z***	4	2	2	2	2
**Radiation type**	CuK_α	CuK_α	CuK_α	MoK_α	CuK_α
**Absorption coefficient, μ/mm^{-1}**	6.421	5.226	3.884	0.467	3.351
**No. of reflections measured**	60632	137009	215009	61516	231685
**No. of independent reflections**	11425	7030	10843	14856	11886
**R_{int}**	0.0630	0.0820	0.0739	- (twinned)	0.0483
**Final R_1 value ($I > 2\sigma(I)$)**	0.0372	0.0530	0.0535	0.0843	0.0438
**Final $wR_2(F^2)$ value ($I > 2\sigma(I)$)**	0.0992	0.1222	0.1488	0.2269	0.1162
**Final R_1 value (all data)**	0.0403	0.0634	0.0577	0.0963	0.0472
**Final $wR_2(F^2)$ value (all data)**	0.1008	0.1277	0.1520	0.2343	0.1207
**Goodness of fit on F^2**	1.053	1.044	1.056	1.125	1.068
**Flack parameter**	-0.0083(16)	-	-	-	-
**Δρ / e Å^{-3}**	1.56 / -0.57	0.88 / -0.51	0.74 / -0.68	1.65 / -0.87	0.72 / 0.1207

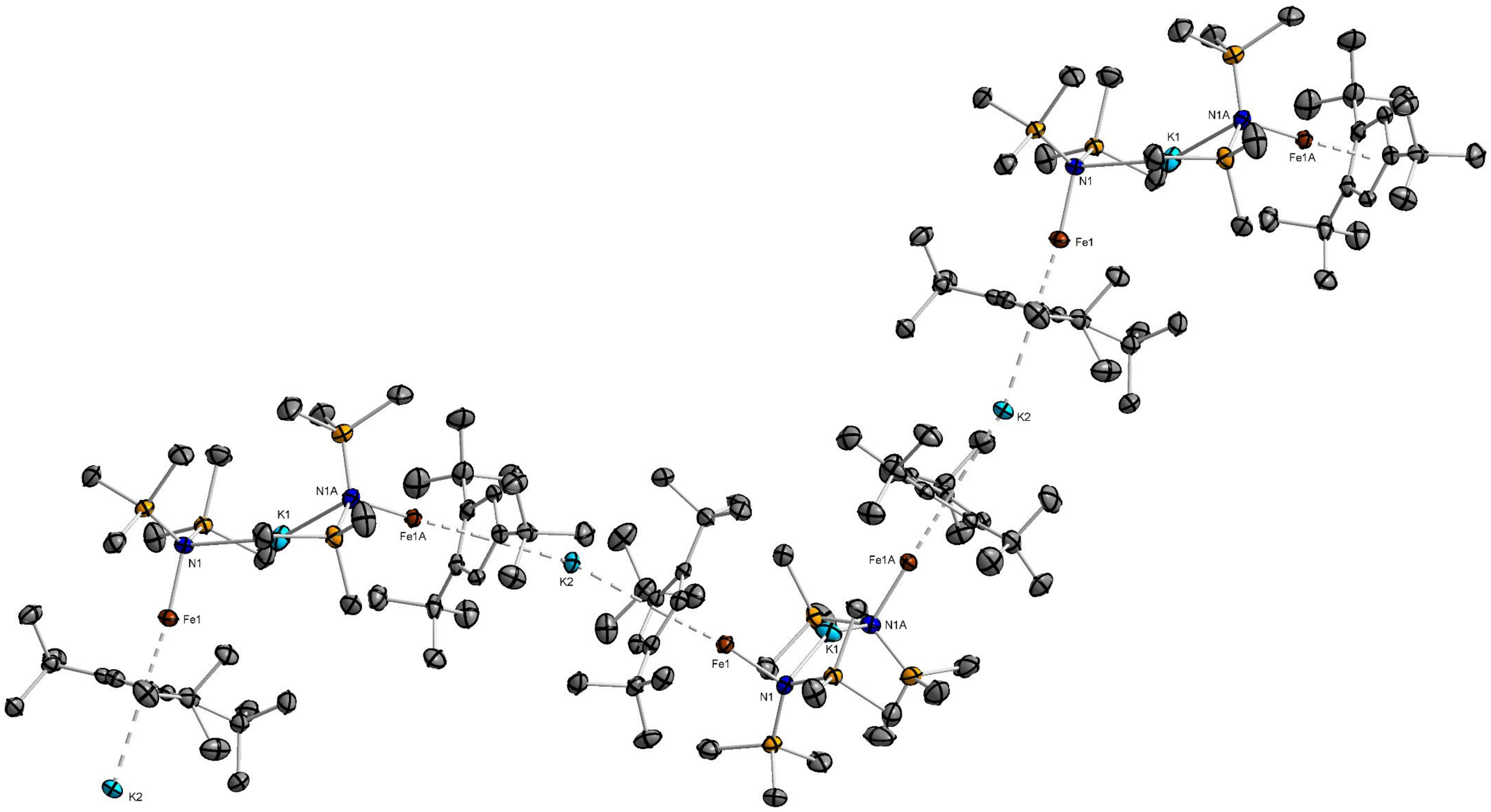

Figure S7. Diamond plot of the solid-state structure of **4**. Ellipsoids are drawn at the 50% probability level. Hydrogen atoms are omitted for clarity.

Table S2. Selected bond lengths (Å) and angles (°) for iron(I) complex **6** at *T* = 80 K.

	**6 (100 K)**	**6 (80 K)**
**Cp$_{cent(A)}$–Fe1(A)**	1.908(1)	1.914(1)
**Fe1–C1(A)**	2.238(2)	2.219(5)
**Fe1–C2(A)**	2.247(2)	2.223(6)
**Fe1–C3(A)**	2.259(2)	2.299(5)
**Fe1–C4(A)**	2.298(2)	2.341(5)
**Fe1–C5(A)**	2.261(2)	2.254(5)
**Fe1–N1(A)**	1.934(2)	1.942(5)
**Fe···Fe**	9.856	9.830
**Cp$_{cent(A)}$–Fe1(A)–N1(A)**	170.7(1)	170.3(2)
**Fe1–N1–Si1**	112.8(2)	119.8(2)
**Fe1–N1–Si2**	118.6(2)	111.3(2)

(a)

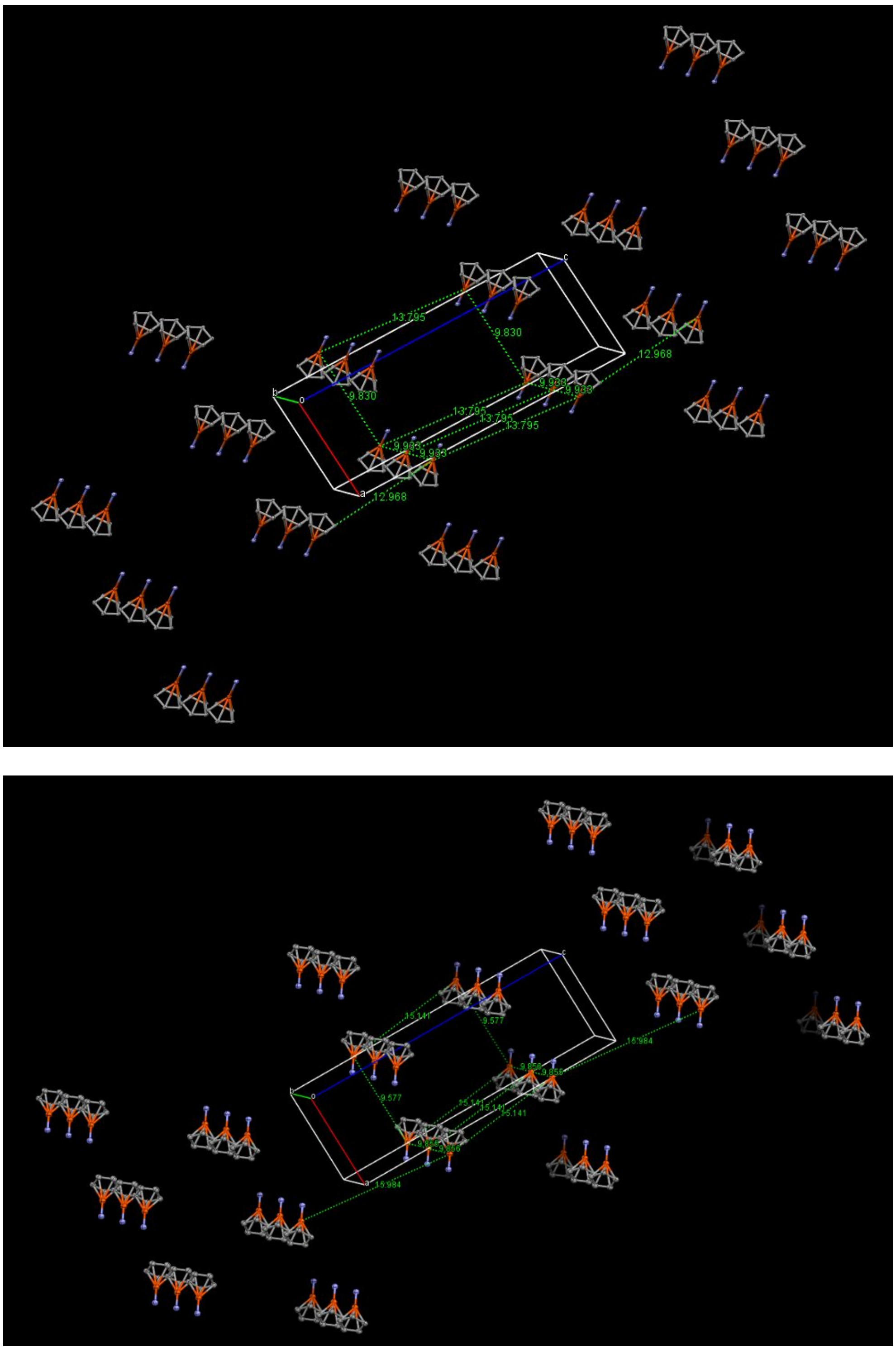

(b)

Figure S8. Comparison of the packing diagrams for **6** recorded at *T* = 80 K (a) and *T* = 100 K (b). [(18-c-6)K]$^+$ cations were omitted for clarity.

3. Zero-field ^{57}Fe Mössbauer Spectroscopy

Table S3. Zero-field ^{57}Fe Mössbauer parameters for compounds **4**–**7** and an identical prepared sample of **4** (denoted as **4***) at T = 60 K (and additionally at T = 20 K for **5**, see Figure S11). The isomer shift (δ) is specified relative to metallic iron at ambient temperatures and was not corrected for the second order Doppler shift.

		T	δ	ΔE_Q	B_{hf}	Γ_{HWHM}	$\upsilon_c = \tau_c^{-1}$	AREA
**4**		60 K	0.76(1)	-1.82(2)	8.9*	0.14*	13.2(6)	87
			0.34(4)	0.46(16)	-	0.48(11)	-	13
	1)	60 K	0.76(1)	-1.70(2)	14.3*	0.14*	31(7)	43
			0.76(1)	-1.90(2)	5.2*	0.12*	6(1)	43*
			0.35(5)	0.34(54)	-	0.62(22)	-	14
**4***		60 K	0.74(1)	-1.90(2)	7.0*	0.20(1)	11(2)	83
			0.43(6)	0.82(10)	-	0.41(10)	-	17
**5**	2)	60 K	0.74(1)	-2.04(2)	-	0.18(1)	-	96
	3)		0.35*	0.34*	-	0.20*	-	4
	4)	60 K	0.74(1)	-2.04(2)	-	0.18(1)	-	96
	3)		0.35*	0.34*	-	0.20*	-	4
		20 K	0.72(1)	-2.08(2)	4.8*	0.19(1)	1.37(17)	91
	3)		0.35*	0.34*	-	0.20(10)	-	9
**6**		60 K	0.74(1)	-1.92(2)	8.3*	0.16(1)	28(7)	86
			0.57(7)	0.90(16)	-	0.41(13)	-	14
**7**		60 K	0.77(1)	-1.82(2)	6.8*	0.15(1)	8(2)	90
			0.87(7)	1.00(22)	-	0.25(13)	-	10
			mm s^{-1}	mm s^{-1}	T	mm s^{-1}	mm s^{-1}	%

*) fixed in the fit; 1) alternative fit with two (crystallographically) non-equivalent ^{57}Fe sites (see discussion in the main text, and Figure S9); 2) here, an integral intensity ratio of A_2/A_3 = 1.5(1) was used for the second (A_2) and third (A_3) absorption line of the magnetic hyperfine pattern (instead of A_2/A_3 = 2, as expected for polycrystalline specimens in zero applied magnetic field), suggesting the presence of texture effects in this sample; cf., Table S4 3) parameters (δ, ΔE_Q) of the impurity contribution were taken from **4**, while the line width was set to the value of the natural line width; 4) an alternative fit with a doublet of Lorentzian lines revealed almost identical parameters and an intensity ratio of 1.2(1) between the two lines of the main signal, indicating that for **5** at T = 60 K the fast dynamic relaxation limit is reached.

Table S4. Zero-field ^{57}Fe Mössbauer parameters at T = 5 and 20 K for compound **5**, alternatively fitted with a fixed integral intensity ratio of A_2/A_3 = 1.5 to account for the texture effects observed at T = 60 K (cf. Table S3). The isomer shift (δ) is specified relative to metallic iron at ambient temperatures and was not corrected for the second order Doppler shift.

		T	δ	ΔE_Q	B_{hf}	Γ_{HWHM}	$\upsilon_c = \tau_c^{-1}$	AREA
**5**		5 K	0.51(3)	-2.18(6)	5.0(2)	0.12(12)	0.00(12)	13
			0.65(2)	-2.24(4)	5.0*	0.22(2)	1.0(2)	77
	1)		0.35*	0.34*	-	0.20*	-	10
		20 K	0.73(1)	-2.06(2)	5.0*	0.18(1)	1.11(10)	96
	1)		0.35*	0.34*	-	0.20*	-	4
			mm s^{-1}	mm s^{-1}	T	mm s^{-1}	mm s^{-1}	%

*) fixed in the fit; 1) parameters (δ, ΔE_Q) of the impurity contribution were taken from **4**, while the line width was set to the value of the natural line width.

Table S5. Zero-field ^{57}Fe Mössbauer parameters at T = 10 K of supplementary measurements on compound **4** and an identically prepared sample of **4** (denoted as **4***). The isomer shift (δ) is specified relative to metallic iron at ambient temperatures and was not corrected for the second order Doppler shift.

		T	δ	ΔE_Q	B_{hf}	Γ_{HWHM}	$\upsilon_c = \tau_c^{-1}$	AREA
**4**		10 K	0.80(1)	-1.66(2)	14.3(1)	0.15(2)	0.00(1)	44
			0.75(1)	-1.86(2)	5.2(1)	0.12(1)	0.03(1)	51
			0.35*	0.34*	-	0.62*	-	5
**4***		10 K	0.83(1)	-1.64(2)	14.6(1)	0.19(10)	0.00(10)	24
			0.74(1)	-1.92(2)	5.2(1)	0.20(2)	0.04(2)	67
			0.43*	0.82*	-	0.41*	-	9
			mm s^{-1}	mm s^{-1}	T	mm s^{-1}	mm s^{-1}	%

*) fixed in the fit.

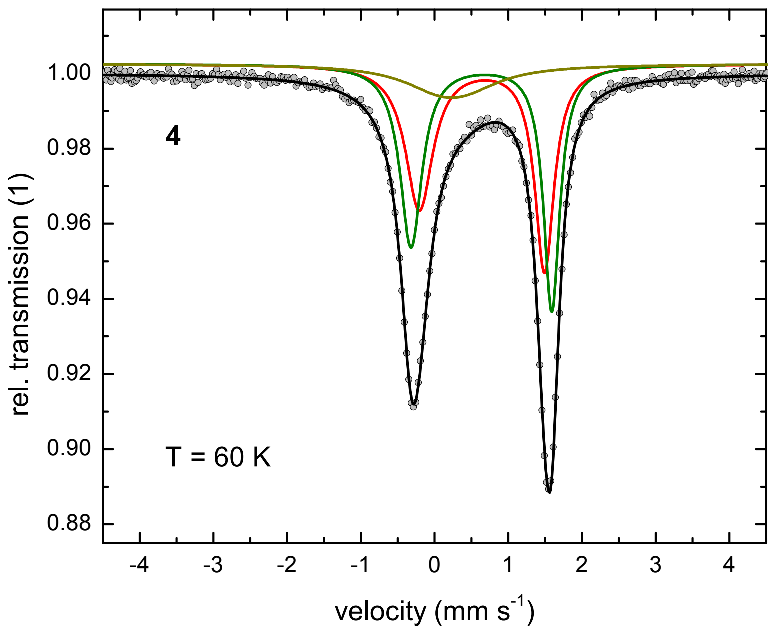

Figure S9. Zero-field ^{57}Fe Mössbauer spectrum of the chain polymer **4** at T = 60 K. Symbols: experimental data (i.e., the same data as shown in Figure S3). Solid lines: two component fit with the Blume-Tjon relaxation model.[1] The black line now represents the superposition of three sub-spectra associated with two (crystallographically) non-equivalent ^{57}Fe sites in **4** (red and green) and the presence of an unidentified impurity (dark yellow). Parameters of the fit are shown in Tables 4 (main text) and S3, details are described in the main text.

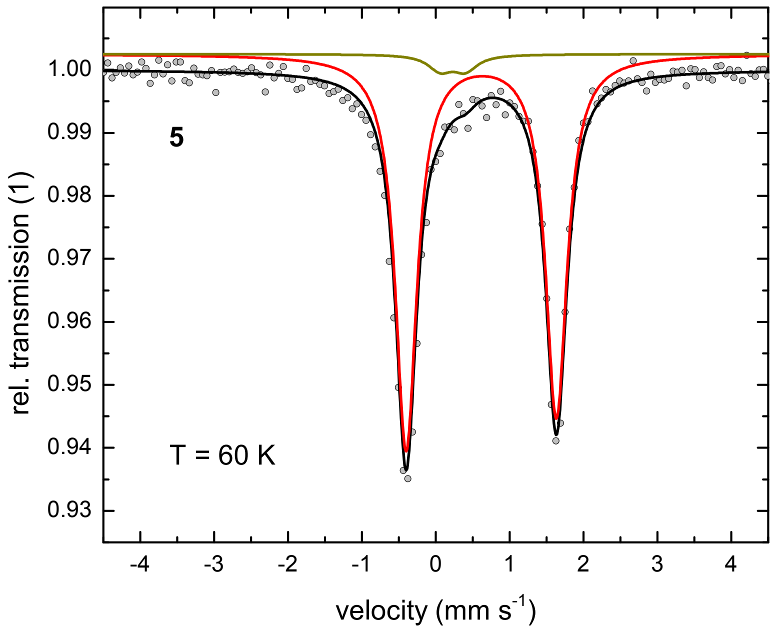

Figure S10. Zero-field ^{57}Fe Mössbauer spectrum of the chain polymer **5** at T = 60 K. Symbols: experimental data. Solid lines: fit with the Blume-Tjon relaxation model.[1] The black line represents the superposition of two sub-spectra associated with a single site (time-averaged) "dynamic" doublet of Lorentzian lines of **5** (red) and the presence of an unidentified impurity (dark yellow). Parameters of the fit are shown in Tables 4 (main text) and S3.

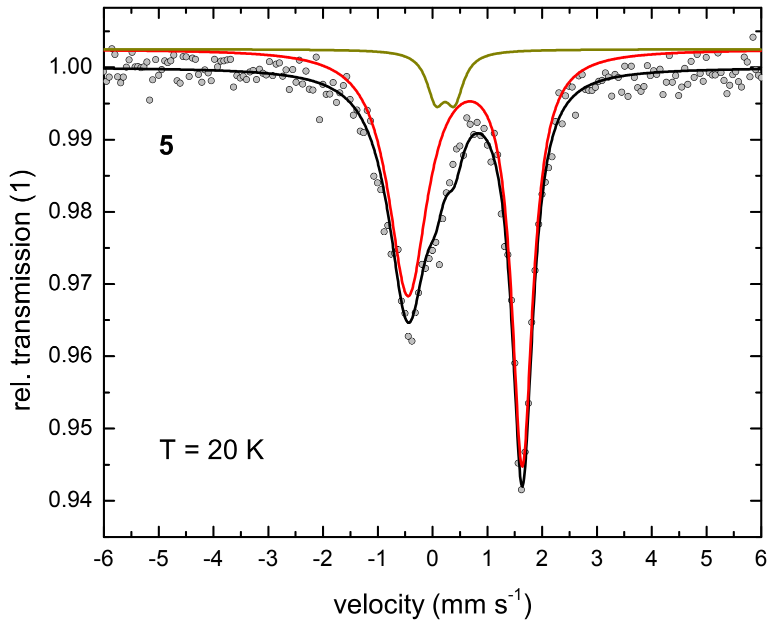

Figure S11. Supplementary zero-field ^{57}Fe Mössbauer spectrum of the chain polymer **5** at T = 20 K. Symbols: experimental data. Solid lines: fit with the Blume-Tjon relaxation model.[1] The black line represents the superposition of two sub-spectra associated with a single site (time-averaged) "dynamic" doublet of Lorentzian lines of **5** (red) and the presence of an unidentified impurity (dark yellow). Parameters of the fit are shown in Tables 4 (main text) and S3.

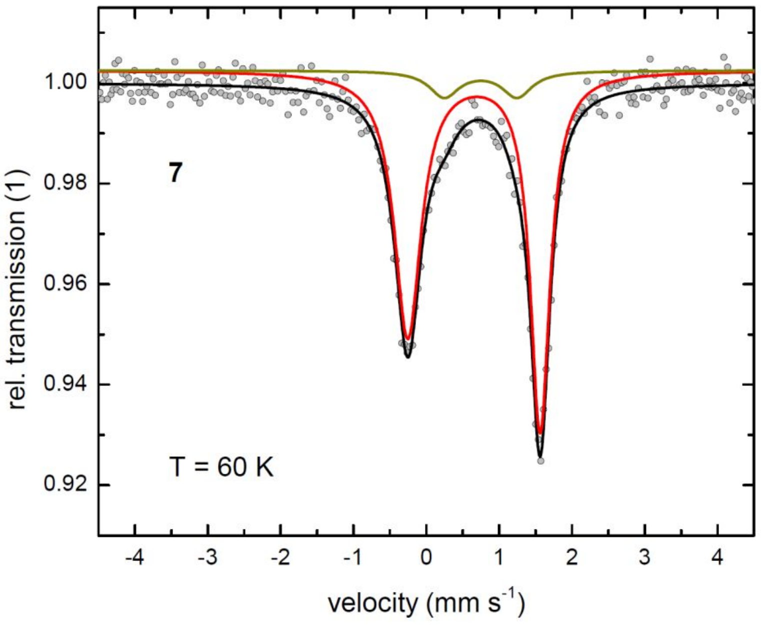

Figure S12. Zero-field ^{57}Fe Mössbauer spectrum of the chain polymer **7** at T = 60 K. Symbols: experimental data. Solid lines: fit with the Blume-Tjon relaxation model.[1] The black line represents the superposition of two sub-spectra associated with a single site (time-averaged) "dynamic" doublet of Lorentzian lines of **7** (red) and the presence of an unidentified impurity (dark yellow). Parameters of the fit are shown in Tables 4 (main text) and S3.

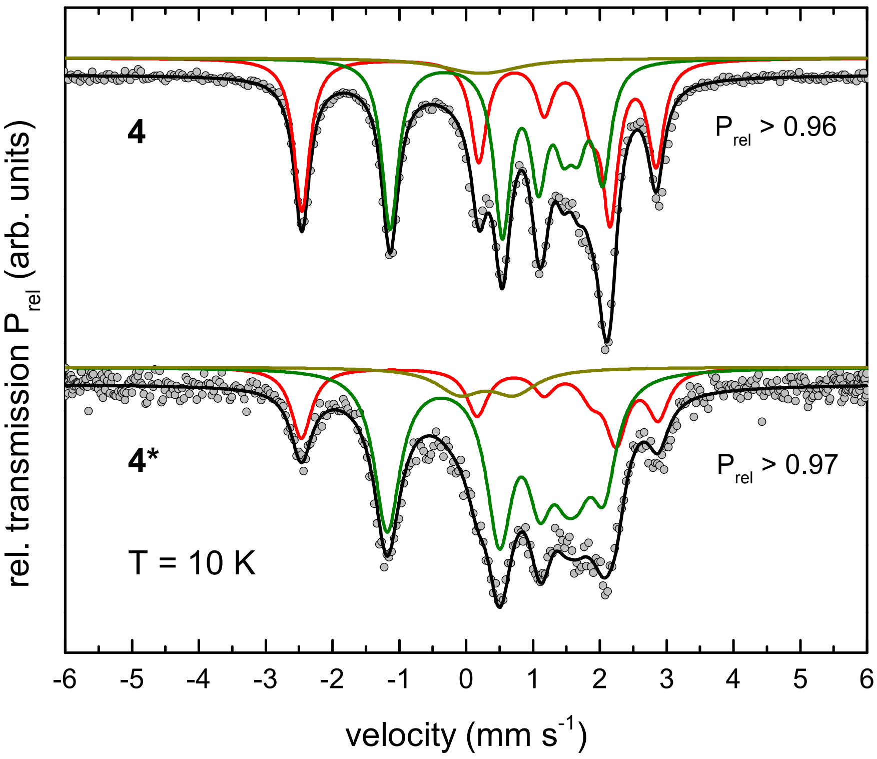

Figure S13. Zero-field ^{57}Fe Mössbauer spectra of the chain polymer **4** and an identically prepared sample of **4** (denoted as **4***) at *T* = 10 K. Symbols: experimental data. Solid lines: fit with the Blume-Tjon relaxation model.[1] The black line represents the superposition of three sub-spectra associated with two (magnetically) non-equivalent ^{57}Fe sites in **4** and **4*** (red and green) and the presence of an unidentified impurity (dark yellow). Parameters of the fit are shown in Table S5, details are described in the main text.

4. Computational Studies

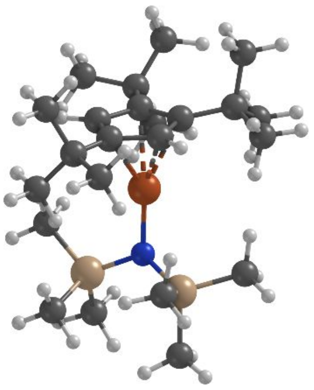

4 (Fe1)

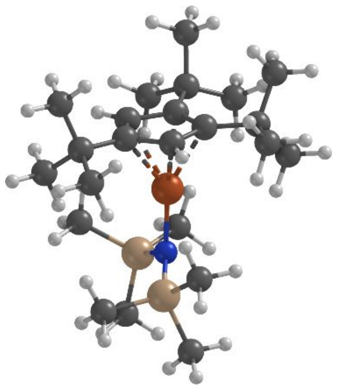

4 (Fe1A)

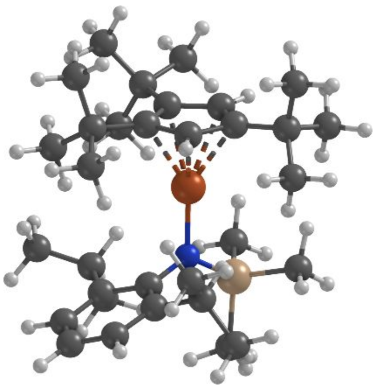

5

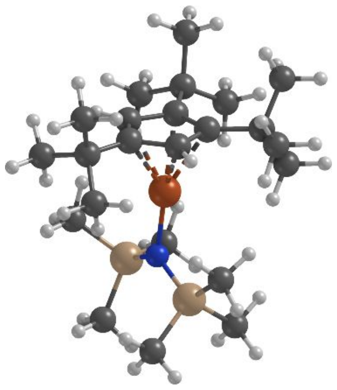

6

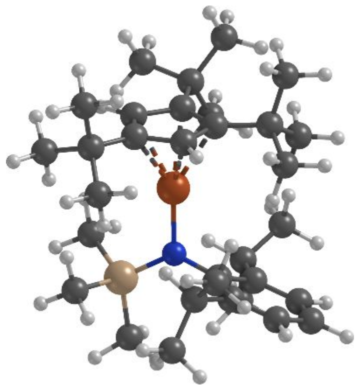

7

Figure S14. Monoanionic model structures of **4**-**7**, used for the computational studies (color code: Fe – orange; N – blue; Si – beige).

Table S6. Calculated electron density ρ at the position of the Fe(I) nucleus and the derived ^{57}Fe Mössbauer isomer shift δ for the complexes **4**-**7** (DFT/TPSSh/TZVP level of theory; δ has been accordingly calculated to reference[2]; $\delta = -0.321(\rho - 11580) + 1.466$)

	ρ (e/a_0^{-3})	δ (mm·s^{-1})
**4** (Fe1)	11582.25391	0.74
**4** (Fe1A)	11582.32686	0.72
**5**	11582.18958	0.76
**6**	11582.26759	0.74
**7**	11582.14950	0.78

Table S7. Electronic charges obtained via natural population analysis (NPA) of selected molecular fragments in the Fe(I) complexes **4**-**7** (DFT/TPSSh/TZVP level of theory; values in parentheses represent the NPA spin population).

	Fe		N		Cp’	
**4** (Fe1)	0.781	(2.896)	−1.750	(0.018)	−1.211	(0.079)
**4** (Fe1A)	0.782	(2.896)	−1.755	(0.016)	−1.216	(0.074)
**5**	0.783	(2.893)	−1.293	(0.018)	−1.216	(0.074)
**6**	0.774	(2.883)	−1.772	(0.025)	−1.208	(0.081)
**7**	0.790	(2.896)	−1.292	(0.014)	−1.218	(0.076)

Table S8. Natural population analysis (NPA) of the valence shell of the Fe(I) centers in the complexes **4**-**7** (DFT/TPSSh/TZVP level of theory).

	**4** (Fe1)	**4** (Fe1A)	**5**	**6**	**7**
4s	0.416	0.414	0.407	0.414	0.394
$3d_{z^2}$	1.720	1.722	1.729	1.714	1.733
$3d_{xz}$	1.096	1.123	1.096	1.127	1.094
$3d_{yz}$	1.105	1.093	1.107	1.096	1.118
$3d_{xy}$	1.585	1.657	1.176	1.314	1.829
$3d_{x^2-y^2}$	1.281	1.193	1.687	1.544	1.027
sum	7.203	7.202	7.203	7.209	7.195

Table S9. Relative CASSCF *ab initio* energies (in cm^{-1}) of all quartet and the twelve lowest doublet states for the Fe(I)-based complexes **4**-**7**

2*S* + 1	**4** (Fe1)	**4** (Fe1A)	**5**	**6**	**7**
4	0	0	0	0	0
	663	858	697	557	822
	7786	8026	7566	7937	7472
	8255	8256	7749	8280	7738
	8637	8864	8540	8596	8511
	9059	9194	9032	9220	9026
	9616	9788	9532	9812	9489
	18848	18752	18419	18953	18528
	20180	20524	20448	20444	20290
	22119	22495	21598	22143	21662
2	15035	14922	14702	14775	14689
	16119	15968	16217	15850	16109
	17338	17394	17051	17260	17120
	17596	17628	17358	17531	17408
	18006	17933	17743	17917	17641
	18125	18250	18052	18046	18042
	18236	18296	18170	18129	18200
	20014	19999	19751	19920	19790
	21776	21972	21471	21886	21491
	21895	22096	21634	21999	21672
	22289	22134	22131	22174	21994
	22402	22348	22492	22241	22436
	…	…	…	…	…

Table S10. Relative CASPT2 *ab initio* energies (in cm^{-1}) of all quartet and the twelve lowest doublet states for the Fe(I)-based complexes **4**-**7**

2*S* + 1	**4** (Fe1)	**4** (Fe1A)	**5**	**6**	**7**
4	0	0	0	0	0
	696	918	719	593	895
	8191	8098	8200	8236	8191
	8878	8558	7966	8669	7883
	7869	8304	8271	7797	8450
	9482	9586	9420	9645	9432
	10535	10632	10222	10753	10107
	16040	16154	15577	16203	15750
	17870	17967	18090	17767	17870
	21078	21584	20516	21399	20604
2	12348	12207	11963	12005	11982
	13480	13321	13499	13183	13425
	14979	15014	14681	14853	14762
	15111	15116	14773	14994	14813
	15580	15478	15227	15405	15127
	15809	15836	15488	15605	15485
	15881	15959	15784	15723	15839
	17450	17420	17079	17313	17197
	20242	20324	19834	20275	19927
	20320	20029	19714	20389	19525
	19462	19645	19440	19262	19440
	19663	19497	19947	19553	19906
	…	…	…	…	…

Table S11. Lowest four CASSCF/CASPT2/RASSI-SO state energies (in cm^{-1}) for the Fe(I)-based complexes **4**-**7**

KD	**4** (Fe1)	**4** (Fe1A)	**5**	**6**	**7**
1	0	0	0	0	0
2	102	81	97	113	83
3	838	1021	856	764	989
4	970	1132	983	907	1103

Table S12. *Ab initio* calculated Cartesian components of the *g* factor and corresponding zero-field splitting parameters for the high-spin (S=3/2) complexes **4**-**7** (S_{eff} = 3/2; g_{av} = (g_x + g_y + g_z)/3)

	g_x	g_y	g_z	g_{av}	D (cm^{-1})	E (cm^{-1})	E / D
**4** (Fe1)	2.007	2.015	2.939	2.320	−50.86	−0.19	0.00
**4** (Fe1A)	2.031	2.033	2.795	2.286	−40.67	−0.23	0.01
**5**	2.009	2.020	2.905	2.311	−48.45	−0.27	0.01
**6**	1.990	1.995	3.014	2.333	−56.58	−0.33	0.01
**7**	2.026	2.037	2.806	2.290	−41.39	−0.51	0.01

Table S13. *Ab initio* calculated Cartesian components of the *g* factor the first two Kramers doublets (KDs) for the complexes **4**-**7** (S_{eff} = 1/2)

		**4** (Fe1)	**4** (Fe1A)	**5**	**6**	**7**
KD1	E_{KD1} (cm^{-1})	0.0	0.0	0.0	0.0	0.0
	g_x	0.022	0.033	0.036	0.035	0.073
	g_y	0.022	0.034	0.036	0.036	0.075
	g_z	8.726	8.335	8.633	8.918	8.364
KD2	E_{KD2} (cm^{-1})	101.7	81.3	96.9	113.2	82.8
	g_x	3.985	4.073	4.008	3.936	4.102
	g_y	3.949	4.008	3.954	3.873	3.970
	g_z	3.199	2.938	3.138	3.355	2.958

5. Magnetic Susceptibility Studies

Table S14: Parameters resulting from the fit of dc magnetization data of **4** – **7** to an S = 1/2 pseudospin formalism based Hamiltonian consisting of a Zeeman term with an axial g-tensor as the only parameter. The values obtained for the spin ground state within an S = 1/2 formalism obtained from the ab initio calculations are given after the slash, respectively (compare Table ST8). *The parameter runs into the lower physically reasonable limit of $g_{\perp}$ = 0 and is therefore not determined by the data.

	$g_{\parallel}$	$g_{\perp}$	residual
**4**	7.509(6) / 8.726	n.d.* / 0.028	0.092
**5**	7.674(7) / 8.633	n.d.* / 0.036	0.146
**6**	9.17(2) / 8.9189	n.d.* / 0.036	0.833
**7**	8.09(3) / 8.364	0.86(7) / 0.074	0.123

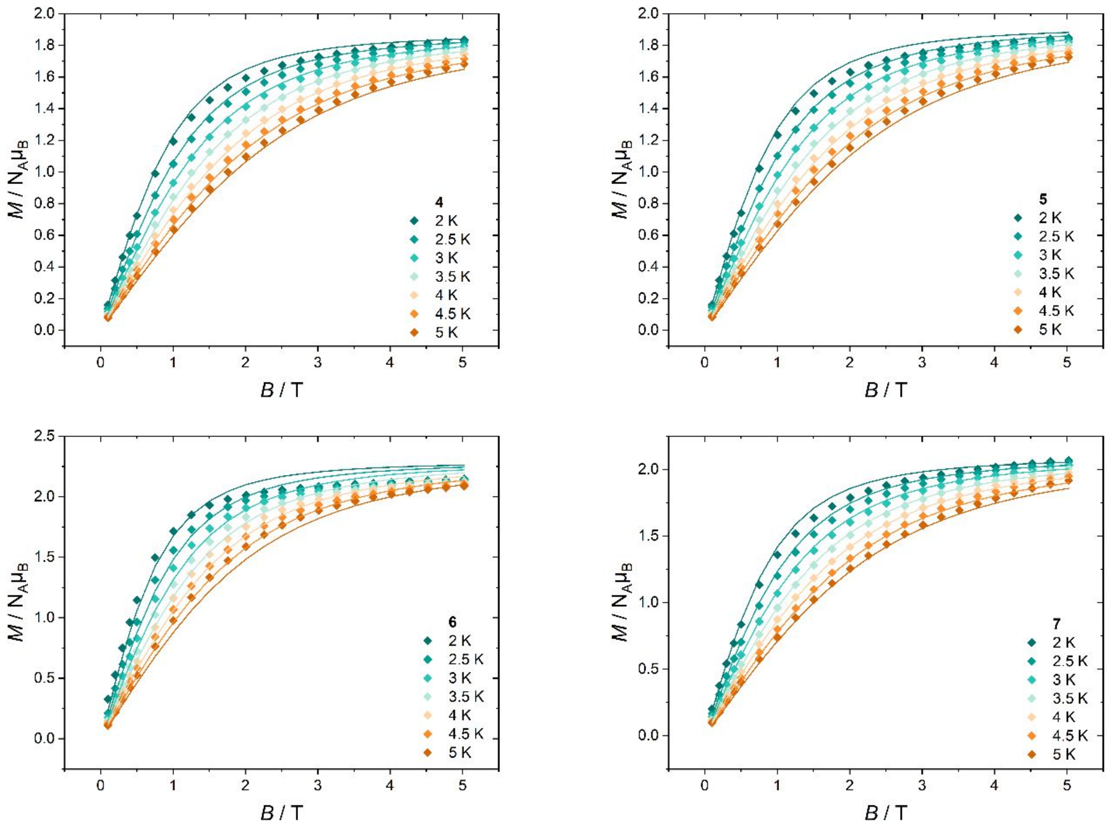

Figure S15: Temperature dependent magnetization (squares) of compounds **4** – **7** to a pseudospin 1/2 formalism (lines, parameters see Table S14).

4 @ H_{dc} = 0 mT

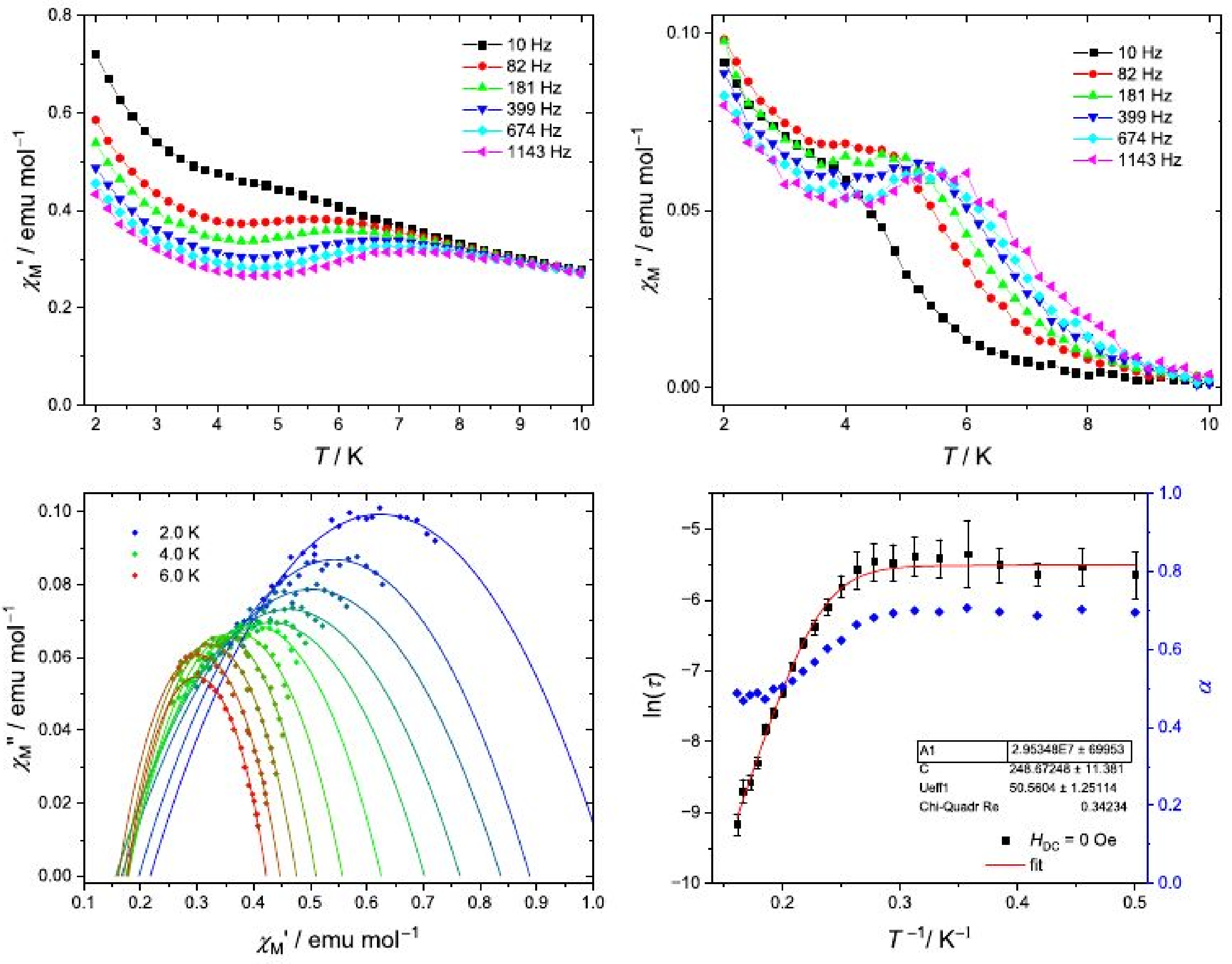

Figure S16: Representation of ac behavior of **4** as the temperature dependency of χ' (left) and χ'' (right) of at H_{dc} = 0 mT, as well as Cole-Cole plots (left bottom) and fitted relaxation data with corresponding dispersion factors α (right bottom).

4 @ H_{dc} = 40 mT

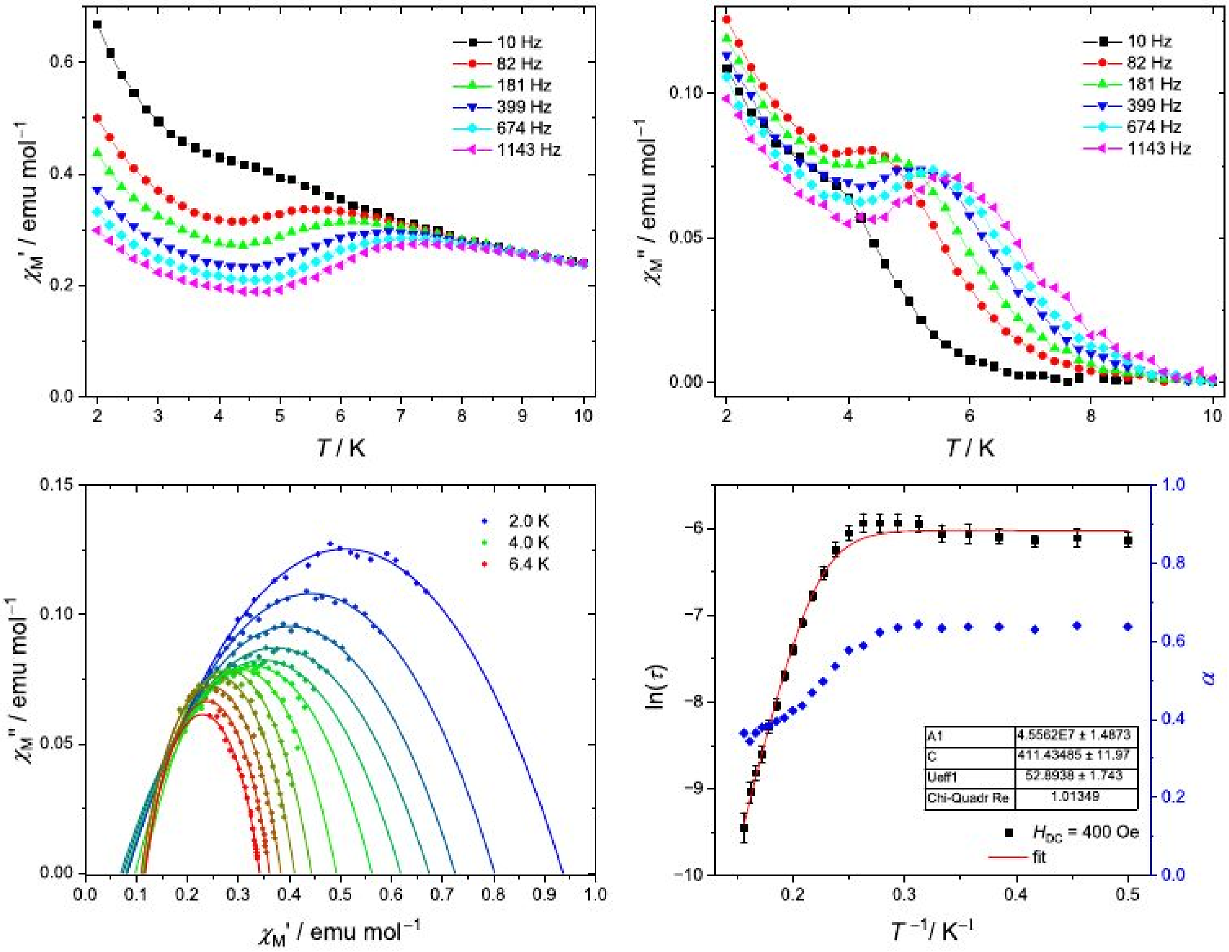

Figure S17: Representation of ac behavior of **4** as the temperature dependency of χ' (left) and χ'' (right) of at H_{dc} = 40 mT, as well as Cole-Cole plots (left bottom) and fitted relaxation data with corresponding dispersion factors α (right bottom).

4 @ H_{dc} = 100 mT

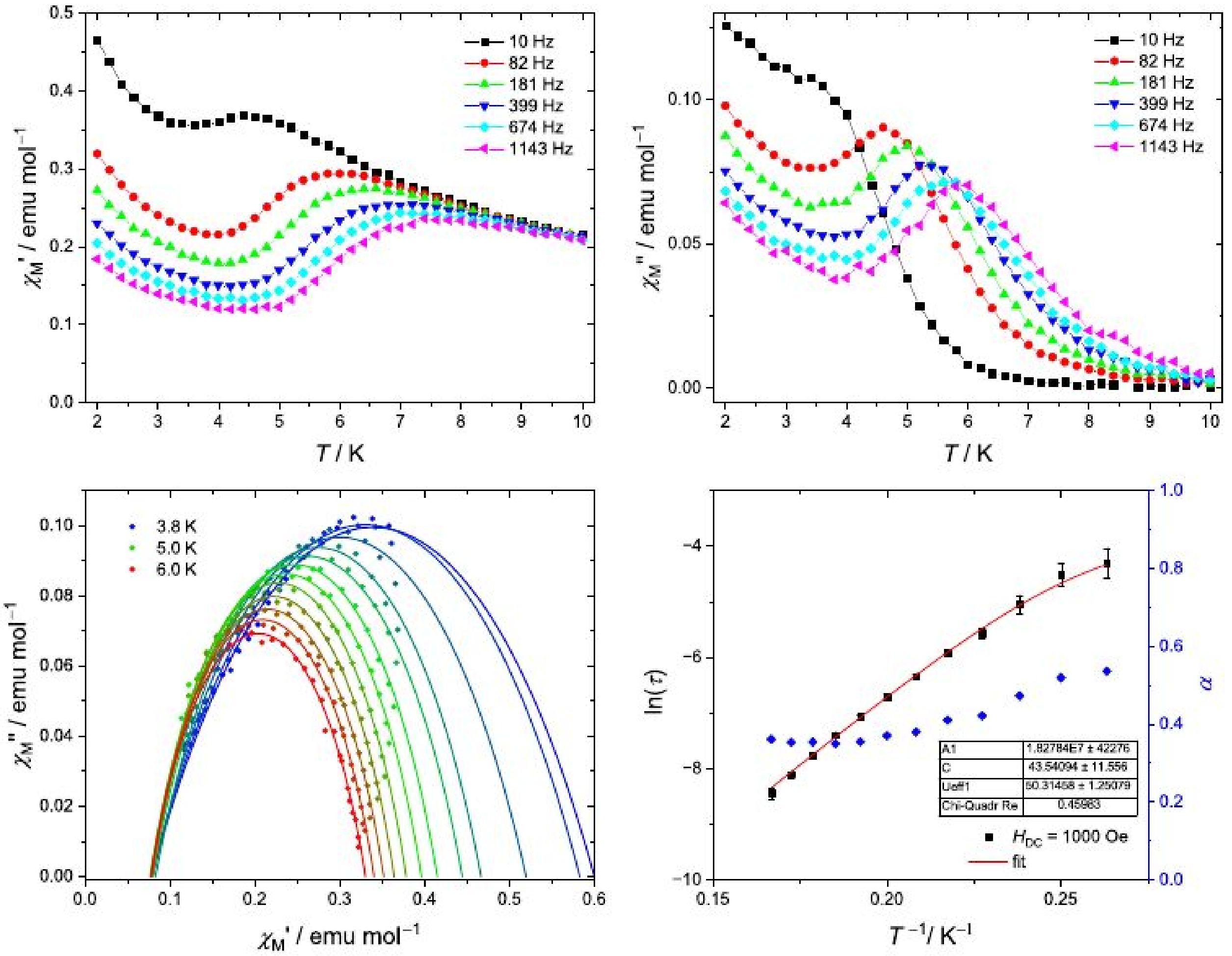

Figure S18: Representation of ac behavior of **4** as the temperature dependency of χ‘ (left) and χ‘‘ (right) of at H_{dc} = 100 mT, as well as Cole-Cole plots (left bottom) and fitted relaxation data with corresponding dispersion factors α (right bottom).

5 @ H_{dc} = 0 mT

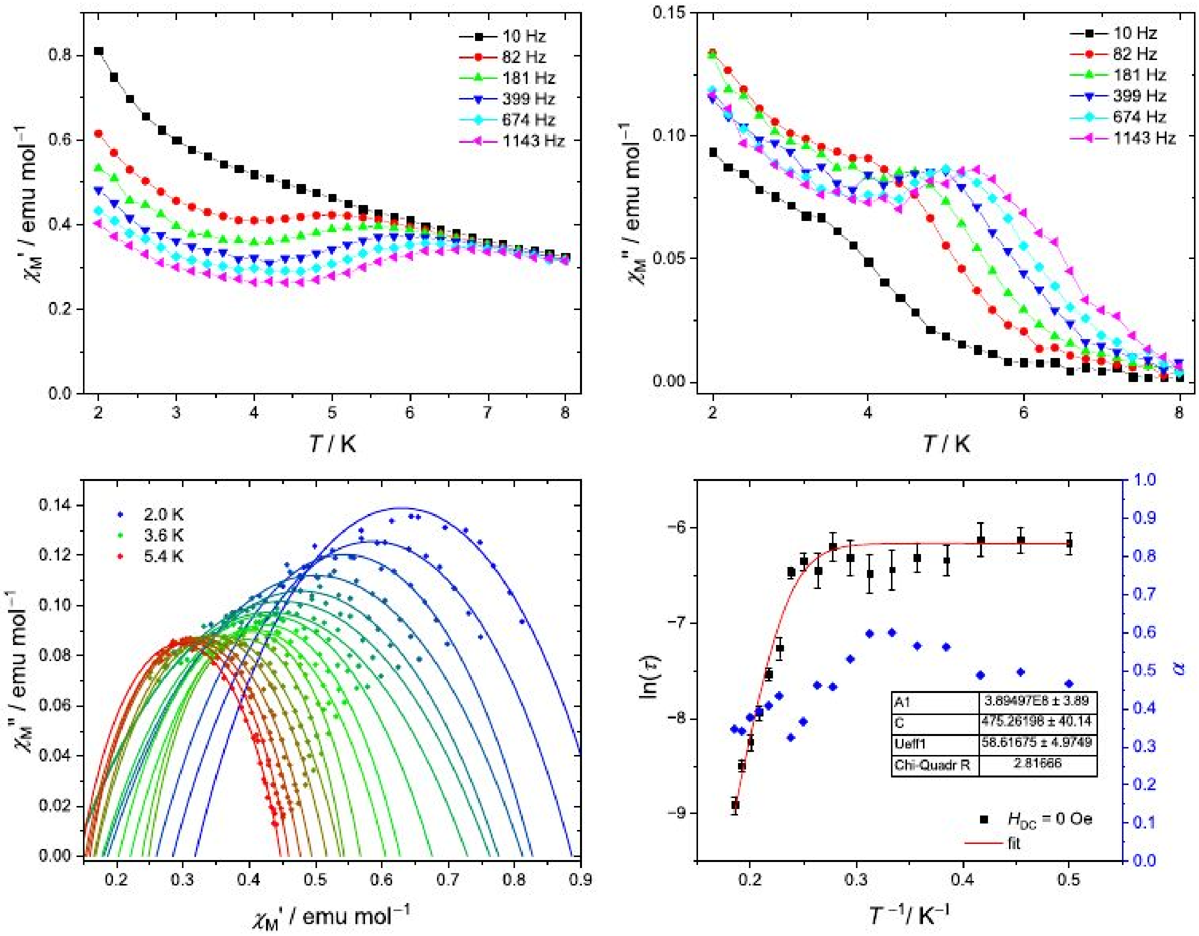

Figure S19: Representation of ac behavior of **5** as the temperature dependency of χ‘ (left) and χ‘‘ (right) of at H_{dc} = 0 mT, as well as Cole-Cole plots (left bottom) and fitted relaxation data with corresponding dispersion factors α (right bottom).

5 @ H_{dc} = 40 mT

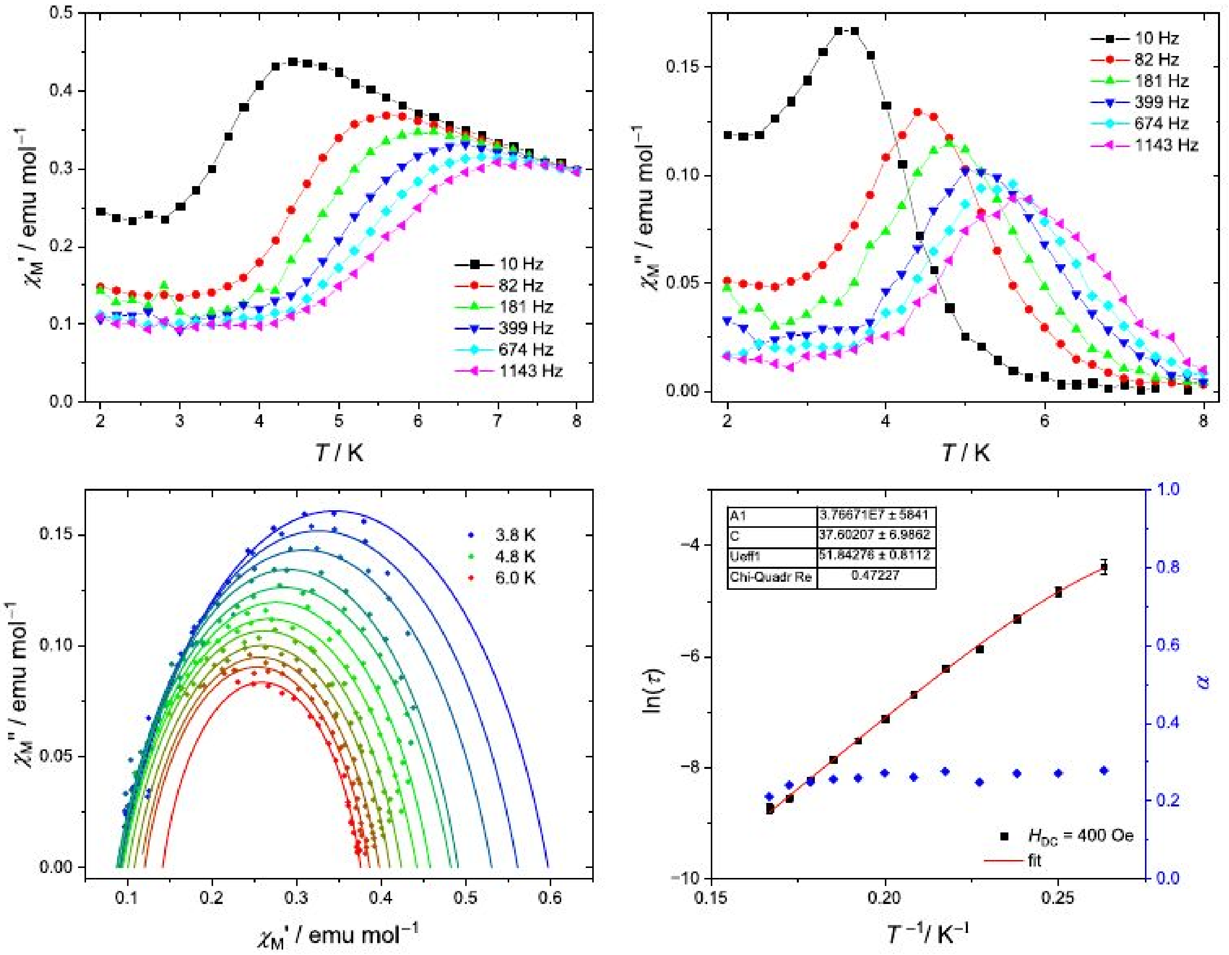

Figure S20: Representation of ac behavior of **5** as the temperature dependency of χ‘ (left) and χ‘‘ (right) of at H_{dc} = 40 mT, as well as Cole-Cole plots (left bottom) and fitted relaxation data with corresponding dispersion factors α (right bottom).

5 @ H_{dc} = 100 mT

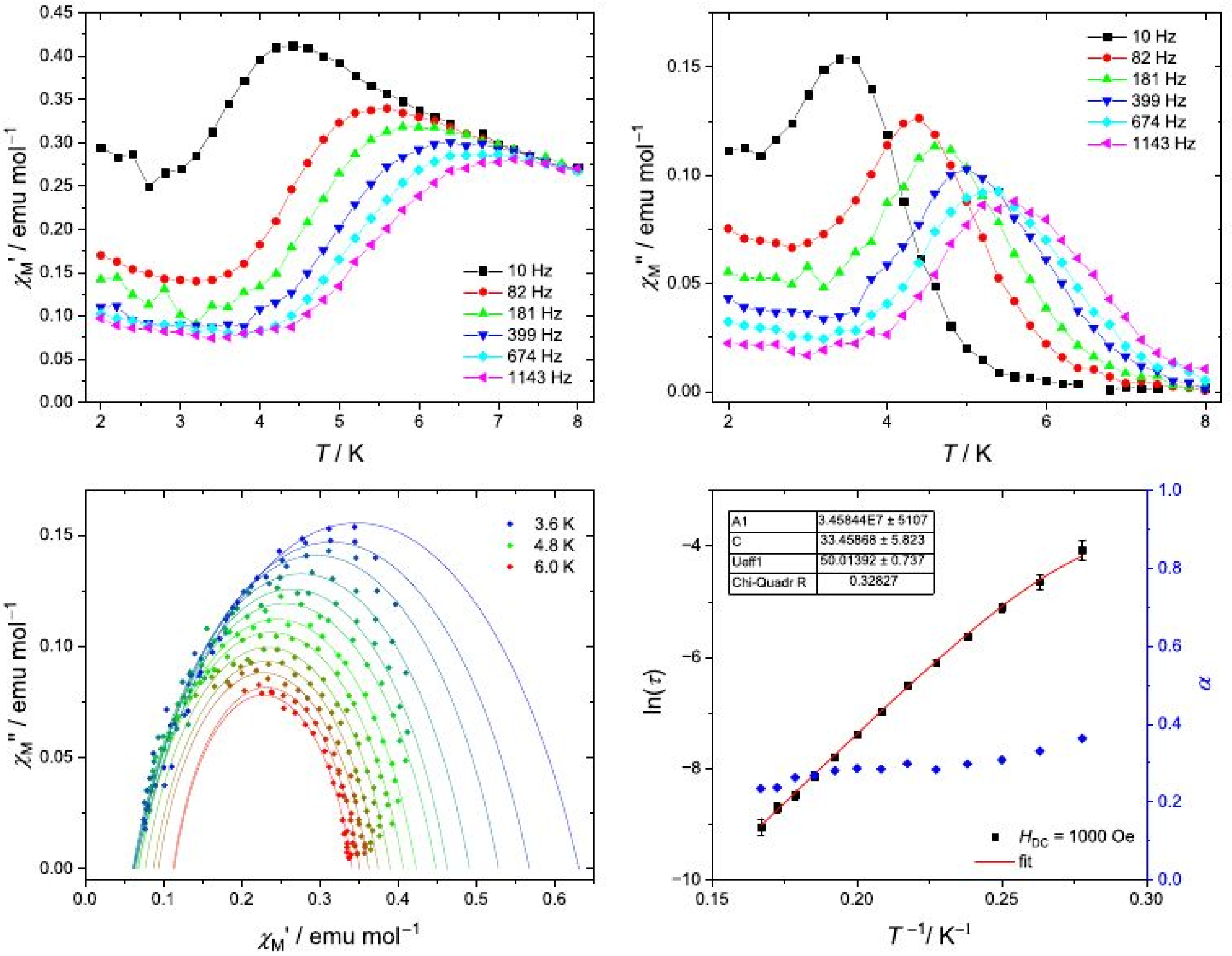

Figure S21: Representation of ac behavior of **5** as the temperature dependency of χ' (left) and χ'' (right) of at H_{dc} = 100 mT, as well as Cole-Cole plots (left bottom) and fitted relaxation data with corresponding dispersion factors α (right bottom).

6 @ H_{dc} = 0 mT

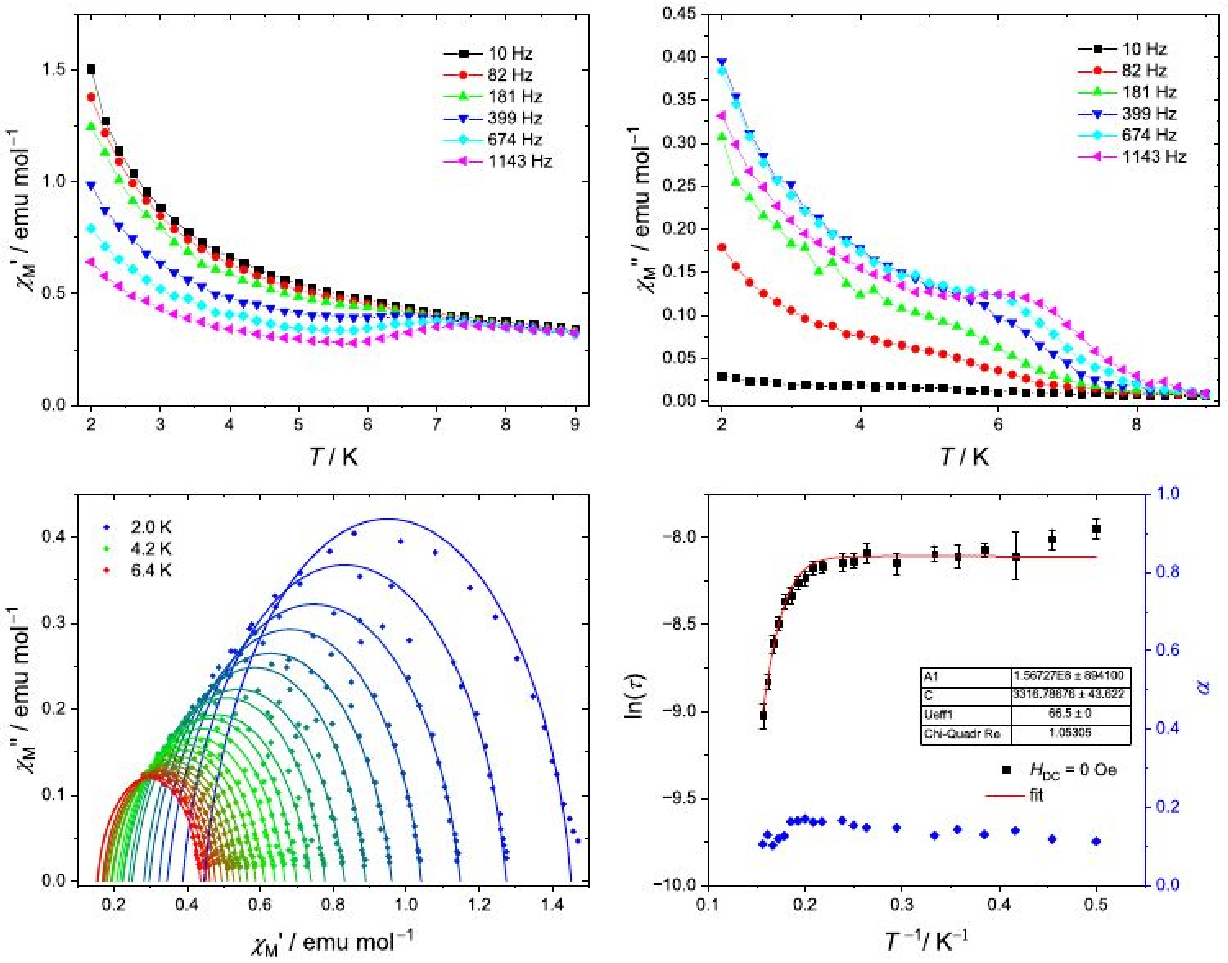

Figure S22: Representation of ac behavior of **6** as the temperature dependency of χ‘ (left) and χ‘‘ (right) of at H_{dc} = 0 mT, as well as Cole-Cole plots (left bottom) and fitted relaxation data with corresponding dispersion factors α (right bottom).

6 @ H_{dc} = 40 mT

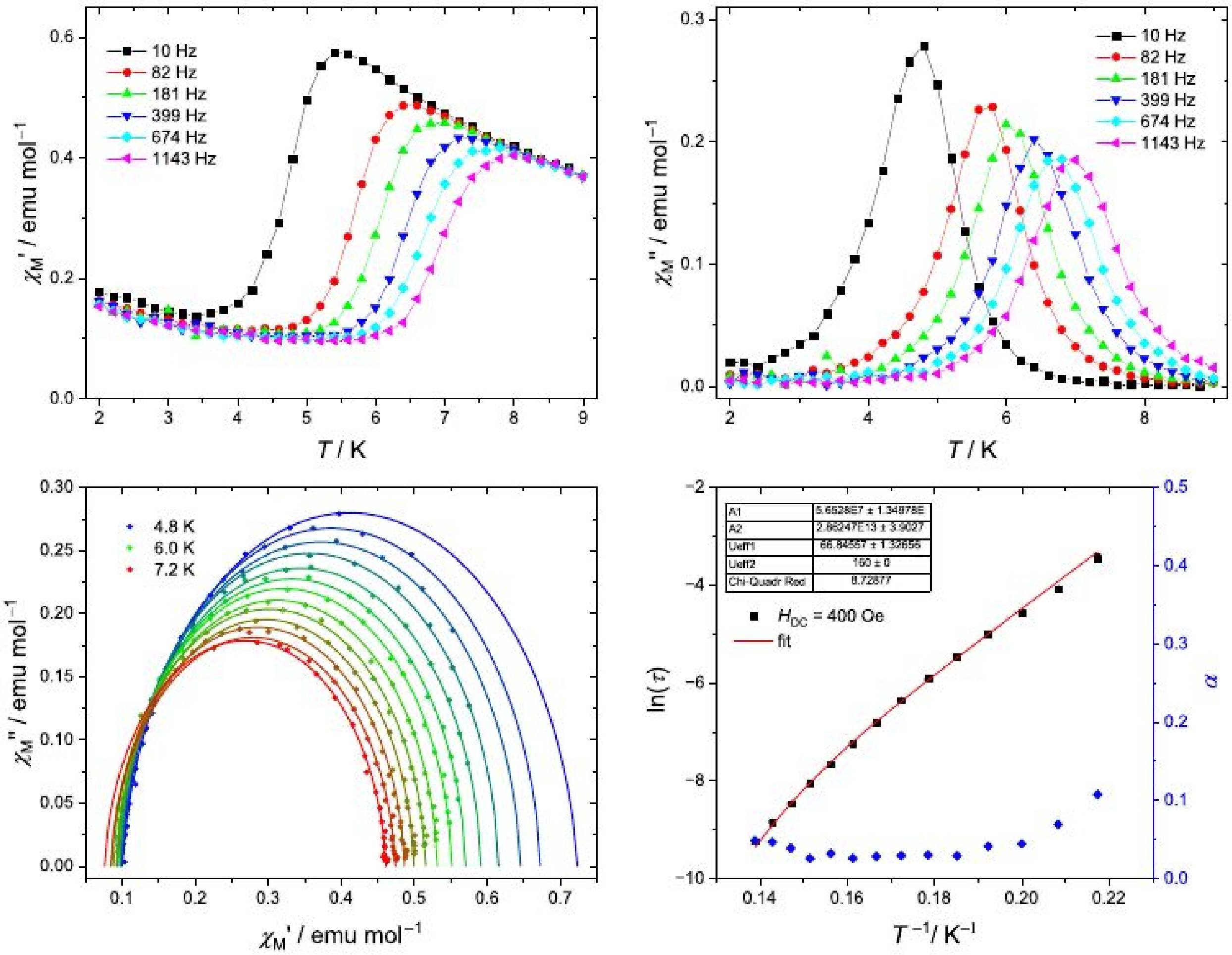

Figure S23: Representation of ac behavior of **6** as the temperature dependency of χ‘ (left) and χ‘‘ (right) of at H_{dc} = 40 mT, as well as Cole-Cole plots (left bottom) and fitted relaxation data with corresponding dispersion factors α (right bottom).

6 @ H_{dc} = 100 mT

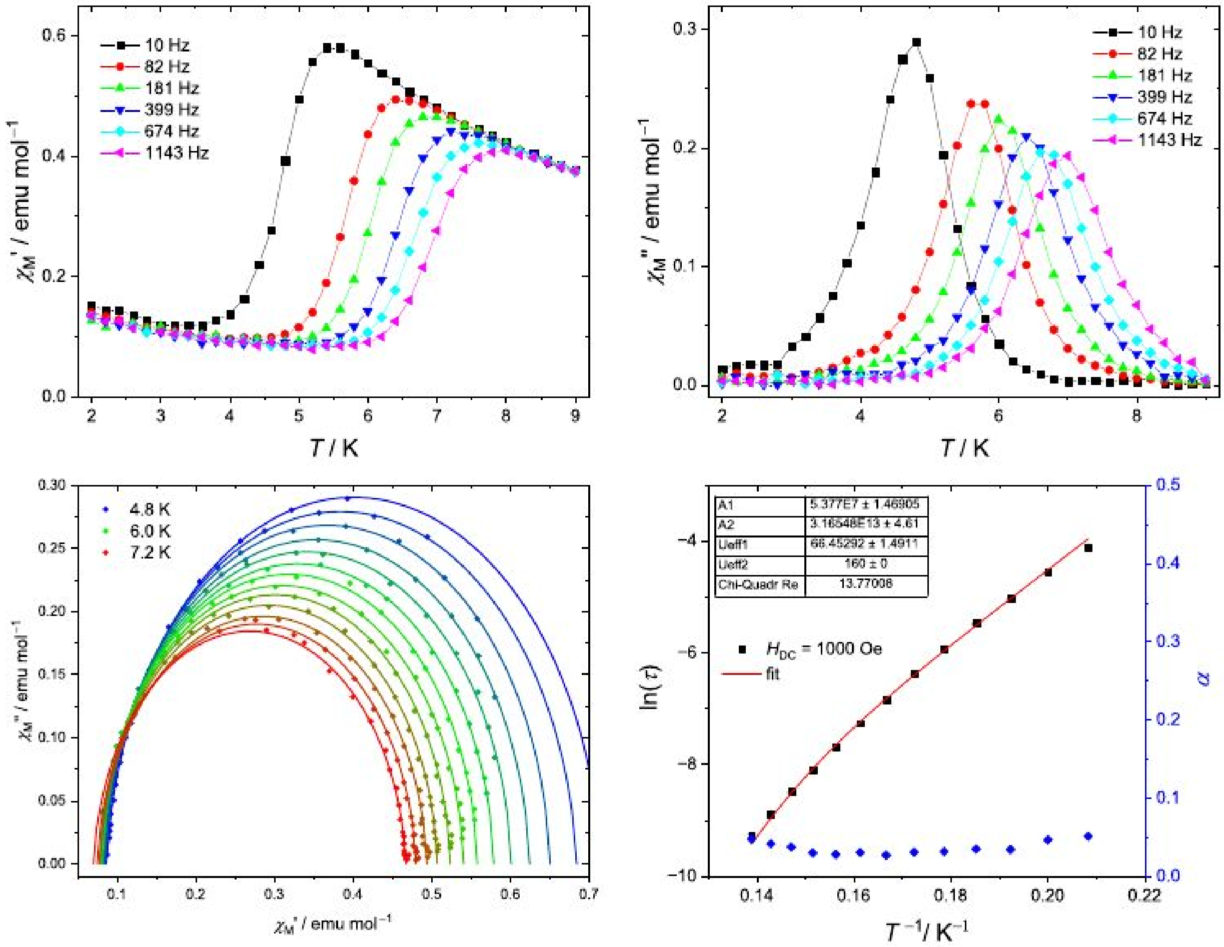

Figure S24: Representation of ac behavior of **6** as the temperature dependency of χ‘ (left) and χ‘‘ (right) of at H_{dc} = 100 mT, as well as Cole-Cole plots (left bottom) and fitted relaxation data with corresponding dispersion factors α (right bottom).

7 @ H_{dc} = 40 mT

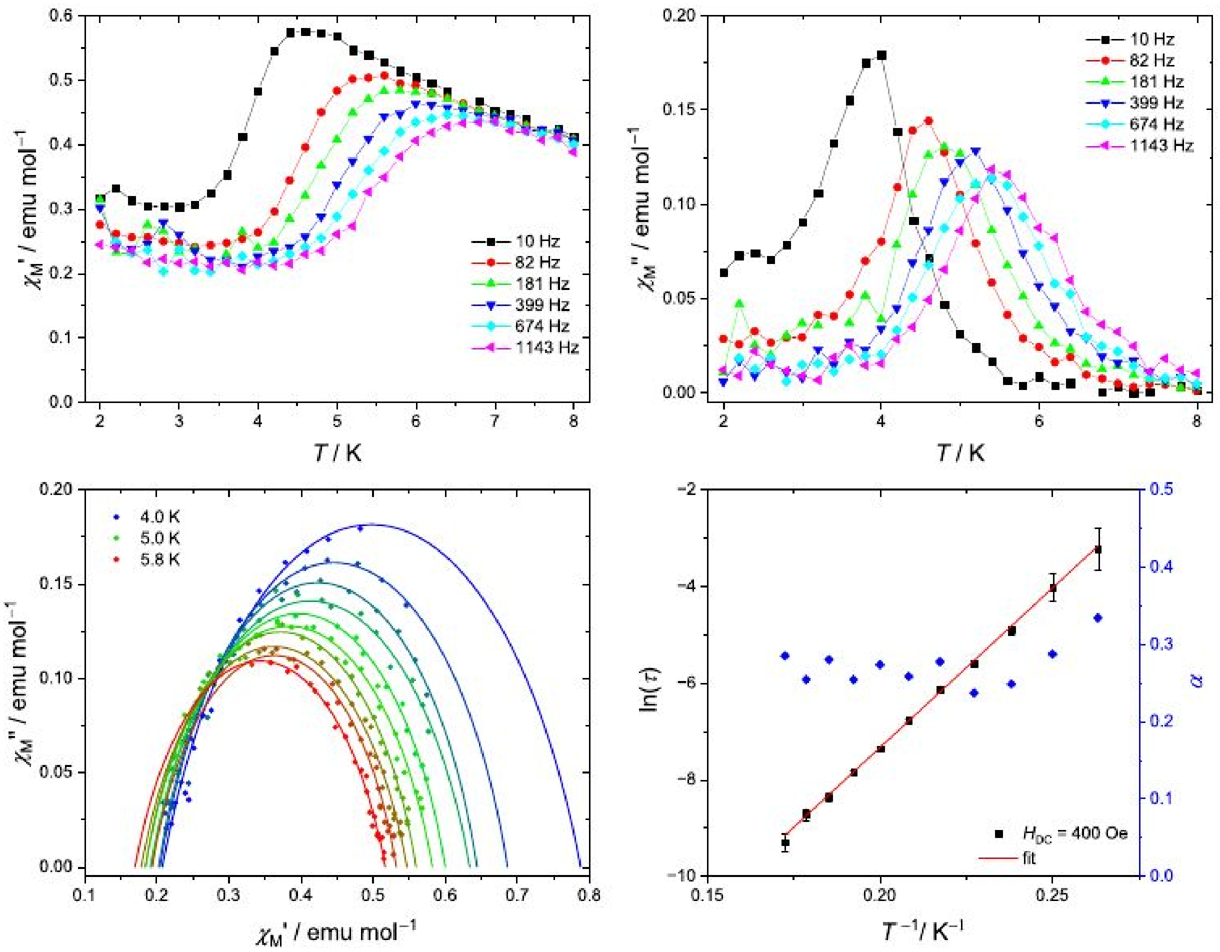

Figure S25: Representation of ac behavior of **7** as the temperature dependency of χ‘ (left) and χ‘‘ (right) of at H_{dc} = 100 mT, as well as Cole-Cole plots (left bottom) and fitted relaxation data with corresponding dispersion factors α (right bottom).

7 @ H_{dc} = 100 mT

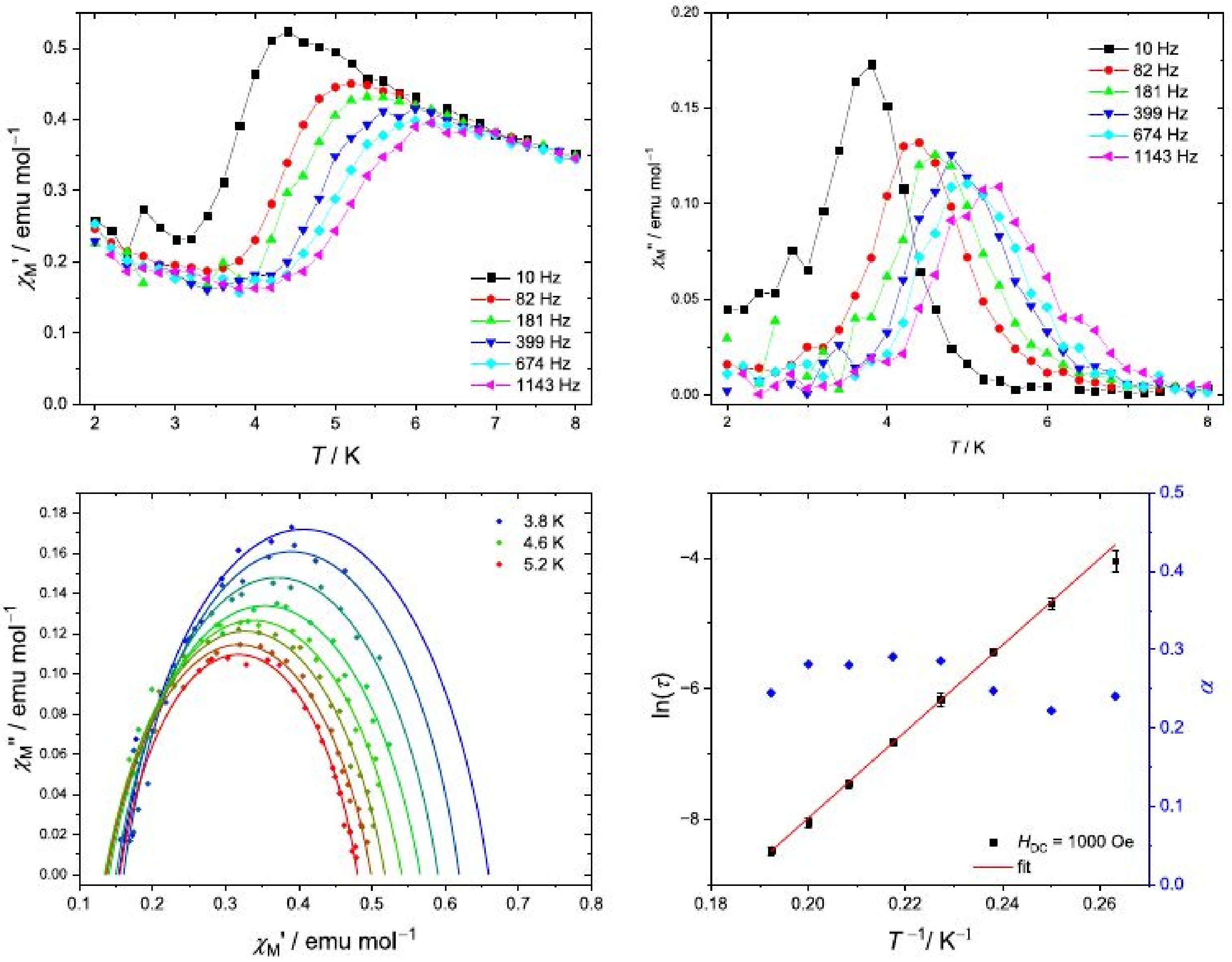

Figure S26: Representation of ac behavior of **7** as the temperature dependency of χ‘ (left) and χ‘‘ (right) of at H_{dc} = 100 mT, as well as Cole-Cole plots (left bottom) and fitted relaxation data with corresponding dispersion factors α (right bottom).

6. Mössbauer extended magnetic relaxation

Table S15: Parameters resulting from the fit of relaxation times τ_C as extracted from AC magnetic susceptibility data of **4** - **7** to eq. (2) in the main manuscript including additional data points obtained from Mössbauer fluctuation rates ν_c at higher temperatures. Parameters marked with an asterisk were fixed in the fit to the values from the fits without Mössbauer data points (U_{vib}, C) described in the main manuscript or from the *ab initio* calculations (U_{Orb}), "(n.d.)" means not determined by the data.

H_{dc}	A_{vib} / s^{-1}	U_{vib} / cm^{-1}	A_{Orb} / s^{-1}	U_{Orb} / cm^{-1}	C / s^{-1}
**4**					
0 mT	2.95(10)·10^{7}	35.2*	7(2)·10^{8}	64(22)	250*
**5**					
0 mT	2.8(2)·10^{8}	39.6(3)	(n.d.)	(n.d.)	475*
**6**					
0 mT	7(6)·10^{7}	43(4)	2(12)·10^{10}	163*	3.32·10^{3}*
**7**					
40 mT	3.2(6)·10^{8}	42.7(6)	(n.d.)	(n.d.)	(n.d.)

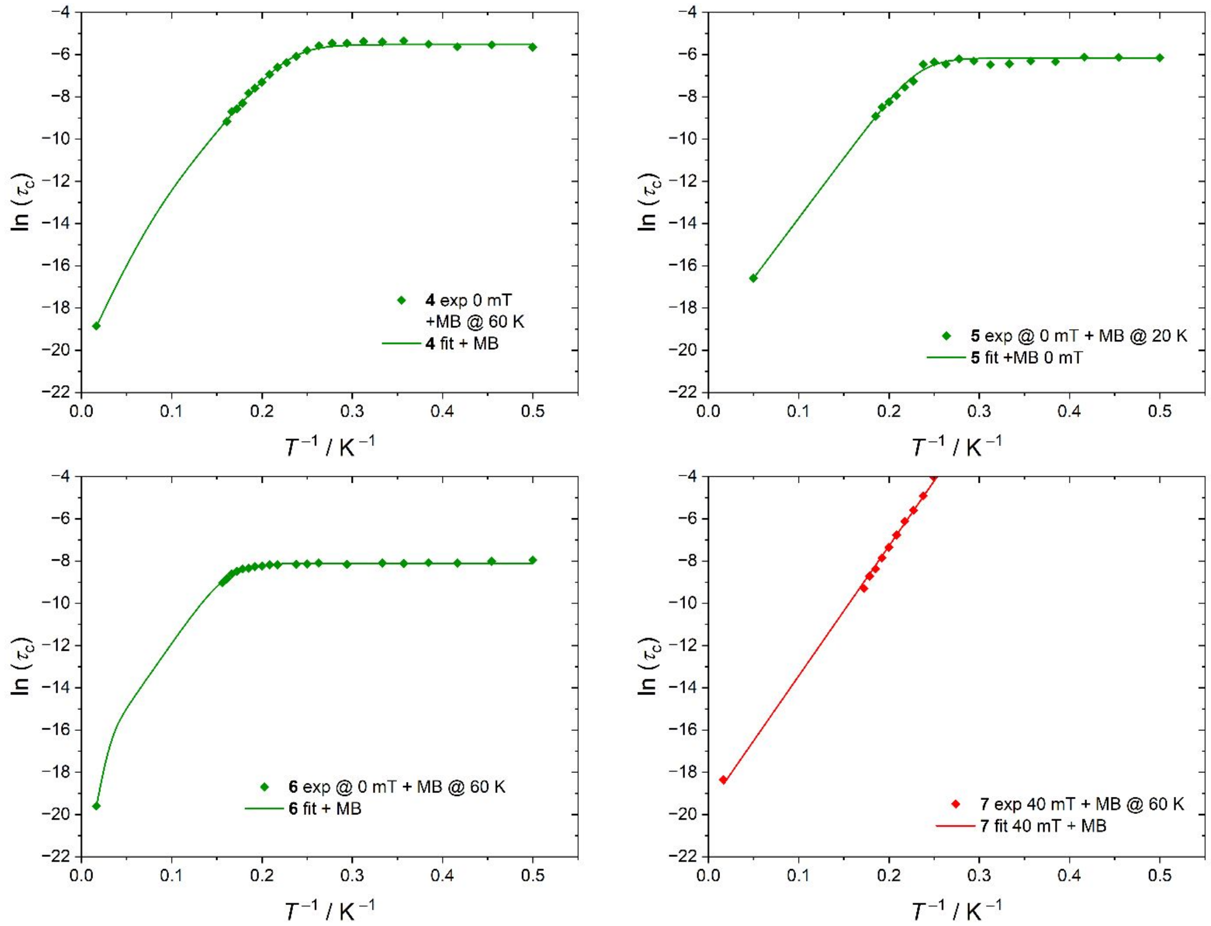

Figure S27: Depiction of the temperature dependence of magnetic relaxation times τ_C for compounds **4** – **7** including the additional points derived from Mössbauer fluctuation rates ν_c at higher temperatures as specified in the legend (compare Long *et al.* [3]). The solid lines represent the corresponding fits to eq. (2) from the main manuscript with the parameters given in Table S15.

7. References

(1) Blume, M.; Tjon, J. A. Mössbauer Spectra in a Fluctuating Environment. *Phys. Rev.* **1968**, *165*, 446-456. DOI: 10.1103/PhysRev.165.446.

(2) Römelt, M.; Ye, S.; Neese, F. Calibration of Modern Density Functional Theory Methods for the Prediction of ^{57}Fe Mössbauer Isomer Shifts: Meta-GGA and Double-Hybrid Functionals. *Inorg. Chem.* **2009**, *48*, 784-785. DOI: 10.1021/ic801535v.

(3) Zadrozny, J. M.; Xiao, D. J.; Long, J. R.; Atanasov, M.; Neese, F.; Grandjean, F.; Long, G. J. Mössbauer Spectroscopy as a Probe of Magnetization Dynamics in the Linear Iron(I) and Iron(II) Complexes $[Fe(C(SiMe_3)_3)_2]^{1-/0}$. *Inorg. Chem.* **2013**, *52*, 13123-13131. DOI: 10.1021/ic402013n.
